# ZnO Nanostructures for Tissue Engineering Applications

**DOI:** 10.3390/nano7110374

**Published:** 2017-11-06

**Authors:** Marco Laurenti, Valentina Cauda

**Affiliations:** Department of Applied Science and Technology, Politecnico di Torino, C.so Duca degli Abruzzi 24, 10129 Turin, Italy; valentina.cauda@polito.it

**Keywords:** ZnO nanostructures, tissue engineering, angiogenesis, antibacterial properties, osteogenic activity, biocompatibility, composite materials

## Abstract

This review focuses on the most recent applications of zinc oxide (ZnO) nanostructures for tissue engineering. ZnO is one of the most investigated metal oxides, thanks to its multifunctional properties coupled with the ease of preparing various morphologies, such as nanowires, nanorods, and nanoparticles. Most ZnO applications are based on its semiconducting, catalytic and piezoelectric properties. However, several works have highlighted that ZnO nanostructures may successfully promote the growth, proliferation and differentiation of several cell lines, in combination with the rise of promising antibacterial activities. In particular, osteogenesis and angiogenesis have been effectively demonstrated in numerous cases. Such peculiarities have been observed both for pure nanostructured ZnO scaffolds as well as for three-dimensional ZnO-based hybrid composite scaffolds, fabricated by additive manufacturing technologies. Therefore, all these findings suggest that ZnO nanostructures represent a powerful tool in promoting the acceleration of diverse biological processes, finally leading to the formation of new living tissue useful for organ repair.

## 1. Introduction

Nowadays, smart biocompatible materials represent one of the most successful approaches in designing artificial scaffolds for tissue engineering (TE) [1]. Scaffolds are conceived as temporary porous structures for promoting new tissue formation and supporting cell growth from the beginning up to the end of the regeneration process [2]. When seeding cells, these must be able to colonize the overall scaffold architecture. Therefore, the cell adhesion on the outer surface as well as their interpenetration within the scaffold network must be promoted. Once adhesion has been obtained, their further proliferation should be also pursued. Finally, a suitable structure within the scaffold framework must be provided, in order to ensure the transport of nutrients and growth factors to the ingrowing cells. All these biological phenomena are strongly dependent on important scaffold parameters, such as the pore size and shape, surface area and topography, as well as the degree of porosity and pore interconnectivity [3]. Other important parameters to be considered are the biodegradation and biocompatible properties of the scaffold materials. Any cytotoxic or immunogenic effect must be avoided. Moreover, the scaffold material should be resorbable or at least inert, depending on the specific application. All these aspects require the use of biocompatible materials, whose re-absorption in the body is governed by a degradation into non-toxic reaction products. As previously mentioned, depending on the target tissue, the scaffold might be completely reabsorbed once the regeneration process is completed. This aspect further highlights the importance of using biomaterials with specific properties, like mechanical and chemical ones. For example, their mechanical strength and chemical stability should be sufficiently good for the original porous architecture to be maintained within the time-frame of tissue regeneration, as well as for mechanically sustaining stresses and loadings generated during the formation of the new tissue. On the other hand, the biomaterial should be also able to degrade in a predictable way, as far as the overall tissue regeneration process is going to be pursued.

A wide plethora of biomaterials, mainly consisting of ceramic materials and polymers, has been successfully tested for the development of biocompatible scaffolds. Ceramics and bioglass matrices certainly represent one of the most investigated categories [4,5,6]. For example, bioglasses are often used for bone TE [5], mainly because of their quick bioactive response that leads to the formation of the mineral phase of bone, i.e., hydroxyapatite, in a very short time [7]. Moreover, a large variety of porous structures, from the macro- to the microscale, can be easily obtained with low-cost synthetic methods [8]. Despite such advantages, bioglass matrices suffer from some major limitations; their degradation process is only partially controllable and the mechanical properties are rather poor. Naturally-derived and synthetic biodegradable polymers also represent a valid solution for TE, as these benefit from ease of preparation and with various synthetic methods [9]. Moreover, the corresponding mechanical and degradation properties can be more easily customized with respect to the target tissue that needs regeneration. Another advantage is their biodegradability. Furthermore, biopolymers often present reactive functional groups on their outer surface. This allows for their easy functionalization with several biological moieties useful for promoting tissue regeneration, like growth factors and pharmaceutical agents [10]. However, biodegradable polymers show hydrophobic behavior in some cases, resulting in a lack of biological sites for cell recognition [11,12]. In other cases, especially for naturally-derived polymers, poor mechanical properties coupled with the expensiveness of raw materials somehow limit their use [12]. Finally, the rate of degradation is not always compatible with respect to the timing required for the tissue regeneration process.

The ease of preparing various micro- and nano-ZnO morphologies showing multifunctional properties opened the way to their investigation for TE as well. Actually, ZnO nanostructures like nanowires (NWs) [13], nanorods (NRs) [14] and nanoflowers (NFs) [15] to name only a few, have been investigated alone, for promoting the adhesion, growth, and differentiation of several cell lines. Additionally, the abovementioned morphologies also exhibited promising antibacterial properties [16]. ZnO nanostructures, featuring antimicrobial activity, osteogenesis and angiogenesis, have been also combined with additive manufacturing technologies such as selective-laser-sintering and 3D printing [17,18,19], with the final aim of designing novel advanced hybrid scaffolds for TE. Actually, several works demonstrated that small percentages of ZnO nanostructures embedded within the main matrix not only resulted in 3D scaffolds with improved mechanical and biodegradable properties, but more interestingly in the rise of new properties due to the presence of ZnO. Thanks to the properties mentioned before, the successful regeneration of living tissues, coupled with antibacterial activity, has been demonstrated in vitro and in vivo, both for pure ZnO nanostructures [15,20] and 3D ZnO-based composite materials [17,18,19,21,22,23,24].

Within this scope, this review highlights the most recent findings in the use of ZnO nanostructures for TE applications. Very promising results have been obtained when ZnO nanostructures are used both in their pure form or combined with ceramic and polymer matrices. In the first paragraph, a general summary of the synthesis methods, properties and applications of ZnO is presented. In the second part, an accurate overview of the most recent findings in using pure ZnO nanostructures for TE will be reviewed. The third paragraph will deal with the use of ZnO nanostructures as fillers in ceramic/polymeric composite systems, and their application to TE. In both cases, i.e., pure ZnO rather than ZnO-based composites, the reviewed findings suggest that ZnO nanostructures may represent a key strategy in the development of new biomaterials with improved functionalities for the promotion of tissue regeneration.

## 2. Zinc Oxide: Synthesis, Properties and Applications

**Figure 1:**
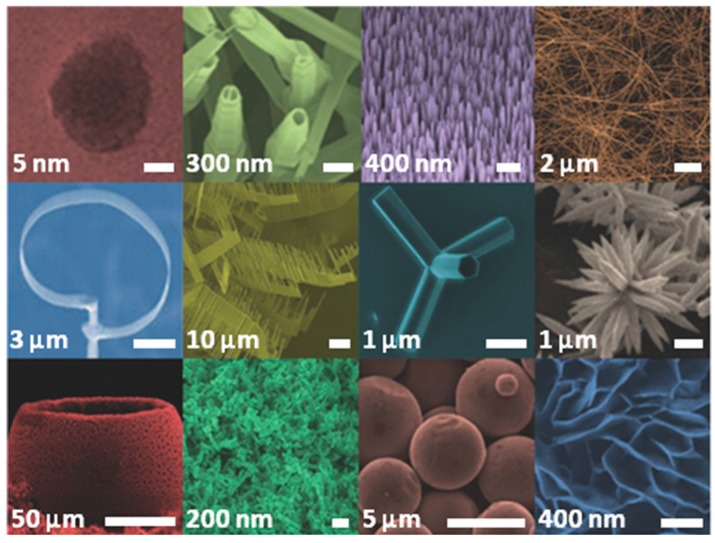
Various and different ZnO nanostructure morphologies. From left to right and from top to bottom: Quantum dot; Nanotubes; Nanowires; Nanobelts; Nanoring; Nanocombs; Tetrapod; Nanoflowers; Hollow spheres; Sponge-like film; Nanosphere; Nanoplates. Adapted with permission from reference [65]. Copyright (2017) John Wiley & Sons, Inc.

Among the metal oxides, zinc oxide (ZnO) is one of the most investigated because of its versatility and multi-functionality. Actually, ZnO on its own possesses very interesting physical and chemical properties that make it a suitable candidate for a huge number of applications, ranging from optics to environmental science, biomedicine and electronics [25,26,27]. Besides that, the versatility of ZnO is also due to the existence of numerous morphologies showing very high specific surface areas [28,29]. These structures, some of which are presented in Figure 1, lie in the micrometer-to-nanometer scale and may include compact and nanobranched thin-film structures [30,31], nanoparticles (NPs) [32], nanowires (NWs) [33,34], nanotubes [35], nanobelts [36], nanorings [37], flower-like morphologies [38], multipods [39] and tetrapods [40]. Additionally, all these ZnO micro- and nanostructures may be very easily prepared by following various synthetic approaches. 

Wet chemical routes [41,42] have been extensively adopted because of their high synthesis versatility, low temperature employed and low costs. For example, ZnO NPs are often prepared by various synthetic processes such as sol-gel [43], hydrothermal [44] and sonochemical [45] ones. Alternatively, green synthesis methods have been also developed, in order to solve some major issues affecting the abovementioned strategies, i.e., self-aggregation and stability of the colloidal suspension [43,46]. ZnO NRs and NWs may be successfully prepared according to hydrothermal routes [47,48,49,50]. In most of the cases, a thin ZnO seed layer (sol-gel derived, sputtered, etc.) is used to start and promote the following nucleation and growth of well-ordered, vertically-aligned ZnO nanostructures [49,50,51]. In this case, the formation of ZnO is obtained through the hydrolysis and condensation of a zinc salt in basic conditions. The seeded substrates are placed in a growing solution; zinc nitrate and zinc acetate are usually employed as zinc precursors, while NaOH, KOH, ammonium hydroxide or hexamethylenetetramine as bases. Such solution-phase syntheses often take place at rather low temperatures (70–90 °C) and for different times (1–24 h). Electrodeposition and template-assisted routes have been successfully applied for the growth of ZnO NWs [41,42]. In the first case, depositions on conductive supports occur in a three-electrode cell operating at low temperatures and in the presence of an electrolyte solution containing the Zn precursor (zinc chloride, zinc nitrate). ZnO may be formed by the reduction of an oxygen precursor, like dissolved molecular oxygen, nitrate ions or hydrogen peroxide; Zn^2+^ and OH^−^ ions react together, leading to the cathodic deposition of ZnO. By changing the applied voltage, time, and precursor concentration, a large variety of hierarchical ZnO nanostructures may be easily obtained [52,53]. Concerning the template-assisted approach, anodic aluminum oxide (with a pore diameter ranging between 20 and 500 nm) and track-etched polycarbonate membranes may be successfully employed as templates [54,55]. Then, the filling of the pores with a ZnO precursor solution may be carried out by wet impregnation or electrodeposition. After that, nucleation of ZnO takes place in the solution diffusing into the template channels, with the ingrowing ZnO crystals adopting the pore morphology and shape.

From the other side, vapor-phase methods such as chemical vapor deposition (CVD) [33], physical vapor deposition (PVD) [49], and epitaxial growth methods [56] are also widely used thanks to the high quality, excellent control and reproducibility over the synthesized ZnO structures and related properties. Moreover, the level of contamination may be minimized, resulting in a high purity of the final material [57]. One-dimensional ZnO NWs may be easily obtained by vapor-liquid-solid phase deposition methods like thermal CVD processes [33,58]. In this case, the presence of a thin ZnO layer or Au NPs is generally required in order to start the nucleation and growth of ZnO nanostructures [33,59]. The formation of ZnO may be obtained by using both ZnO powders or metallic Zn foils, coupled with the addition of oxygen in the gas stream. Various ZnO morphologies may be obtained by using different mixtures of Zn and ZnO sources, oxygen gas pressures and growth temperatures. The growth of vertically-oriented ZnO structures by PVD methods like pulsed laser deposition (PLD) and sputtering have been also demonstrated. Vertically-oriented ZnO nanostructures by PLD are pursued by forming ZnO NPs or a thin layer during the early stage of the deposition process [60,61]. Then, such NPs act as nucleation sites for the growth of vertical ZnO nanostructures [62]. The presence of a thin copper layer acting as a seed was instead reported to be mandatory for growing ZnO NWs by sputtering [63,64]. On the other hand, sputtering resulted in a very promising way to get nanobranched ZnO structures, showing a porous network with a high specific surface area [31]. Such porous structures may be obtained by a two-step process, which involves the deposition of a metallic Zn layer and its further oxidation.

More in details, ZnO is a semiconductor of group II–VI, featuring both ionic and covalent behavior. Most of its properties, like the semiconducting and optical ones, are due to the presence of large band gap (3.37 eV) and exciton binding energy (60 meV) values at ambient temperature [66,67], absorption of ultraviolet (UV) light in the range 200–350 nm and emission in the near UV-visible range from 500 to 600 nm. On the other hand, ZnO is a well-known pyro- and piezoelectric material. In these cases, the central role is played by the crystalline structure. ZnO can exist in three different crystalline frameworks: rock salt, zinc blend and wurtzite [28]. At ambient pressure and temperature, the wurtzite phase is the most thermodynamically stable and therefore the most common one. It is formed by tetrahedral unit cells where the zinc atom is at the center and the oxygen atoms at the four corners. All the tetrahedral units are stacked and oriented in one direction, producing the wurtzite hexagonal symmetry. This non-centrosymmetric and anisotropic crystalline framework makes possible ZnO piezo- and pyro-electricity. The application of an external stimulus, i.e., mechanical stress or temperature, leads to a deformation of the tetrahedron structure of wurtzite ZnO. In this way, the positive and negative centers of charge no longer coincide, inducing the formation of a dipole moment [68].

The combination of the abovementioned physical and chemical properties within high surface area micro/nanostructures make ZnO a potential candidate for a huge number of applications. For example, the biosafety of ZnO has been universally recognized by the Food and Drug Administration, which classified it as a “GRAS” (generally recognized as safe) substance [69]. This aspect, coupled to the optical absorption properties of ZnO, was exploited for the preparation of many commercial formulations for cosmetics and healthcare, including baby- and sun-creams [69]. ZnO is also used as protective, anti-corrosion layer in galvanized steel products [70,71]. Due to the electrical and photoresponsive properties, ZnO has been also integrated into various electronic and optoelectronic devices, such as UV photodetectors [72], transistors [73] and light emitting diodes [74]. The combination of the piezoelectric and semiconducting properties also opened the way to the use of ZnO nanomaterials as nanogenerators for energy harvesting applications [75,76,77,78]. Similarly, the semiconducting and electron transport properties of ZnO nanostructures, combined with their high surface areas, have been used for the fabrication of anodes for Li-ion batteries [58,79], photoanodes in dye-sensitized solar cells [80,81] and in photoelectrochemical cells for water splitting applications [82,83]. Finally, ZnO nanostructures have been successfully proposed in the biomedical field for the fabrication of biosensors [26,84,85]. Actually, the electrical properties of ZnO may be dramatically affected by the adsorption/desorption of specific biomoieties on their surface. Moreover, such high surface areas are prone to be easily functionalized [26]. In this way, the sensitivity of ZnO-based biosensors against targeted biomolecules may be highly improved. 

## 3. Biocompatible and Antibacterial Properties of Pure ZnO Nanostructures

One of the first mechanisms representing the promising application of ZnO nanomaterials for TE deal with the beneficial effects arising from the interaction of living cells with nanostructured ZnO surfaces [86]. Human osteoblasts were cultured for different times (from 1 h up to 21 days) on microphase and nanophase ZnO ceramic compacts, prepared by sintering commercial ZnO powders. The resulting cell behavior was compared and related to each specific ZnO surface area and morphology. The adhesion and activity of osteoblast cells significantly improved in the presence of ZnO surfaces featuring nanometer-sized morphology. When normalized to the corresponding nominal surface areas, osteoblast density, alkaline phosphatase activity (ALP) and calcium mineral deposition significantly improved, respectively, around 171%, 58% and 33% when compared to that obtained for microphase ZnO. Such promising results were ascribed to the increased surface area of nanophase ZnO rather than the microphase ZnO. Indeed, more active sites on the nanophase ZnO surface could be expected, which highly promoted protein interaction during the initial steps of osteoblast adhesion and led to the improved function of the cultured osteoblasts. The combination of both enhanced osteoblast functions and the reduction of the bacterial colonization of nanophase ZnO suggested their possibile and promising application for improving the performance of orthopedic implants. 

The biocompatibility and biosafety of one-dimensional ZnO nanostructures was reported thereafter [87]. ZnO NW arrays were grown on alumina substrates by a vapor-liquid-solid process, removed from the substrate after the growth, and finally dispersed in sterile phosphate buffer solution (PBS) at various concentrations. The biocompatibility assay was performed by using two different cellular lines: tumoral HeLa cells, i.e., a kind of epithelial cell derived from uterin cervix, and L-929 cells, derived from the subcutaneous connective tissue of a mouse. The cultured cells were incubated up to 48 h in the presence of four different ZnO NWs concentrations: 0.1, 1, 10 and 100 μg·mL^−1^. 

**Figure 2:**
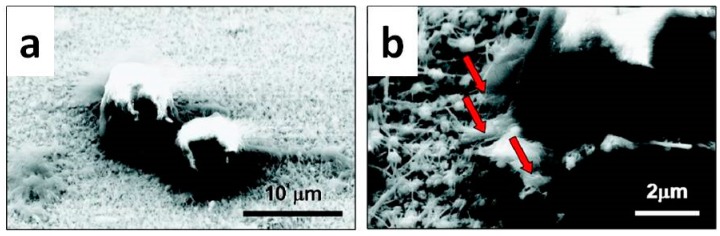
Scanning electron microscopy (SEM) images of HeLa cells on ZnO nanowire (NW) arrays. (**a**) Two HeLa cells grown on the surface of ZnO NW arrays; (**b**) Cells are upheld by the nanowires (NWs). Some ZnO NWs are phagocytosed into the HeLa cell (pointed out by the red arrow). The diameter and the length of the nanowires are ~100 nm and ~1.5 μm, respectively. Adapted with permission from reference [87]. Copyright (2008) American Chemical Society.

**Figure 3:**
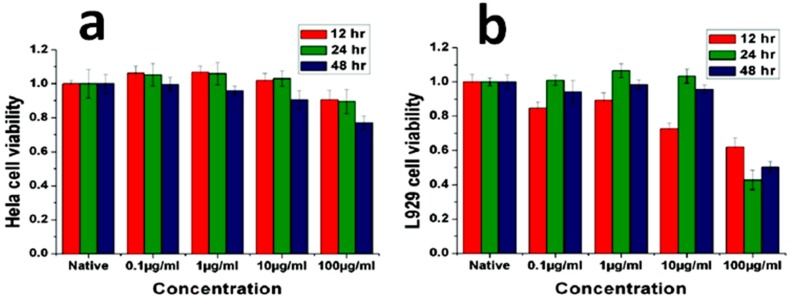
Cell viability tested by the MTT (3-(4,5-dimethylthiazol-2-yl)-2,5-diphenyltetrazolium bromide) method as a function of ZnO NW concentration and time. (**a**) Cell viability of the HeLa cell line in the MTT test, cultured with different concentration of ZnO NWs for 12 h, 24 h, 48 h; (**b**) Viability of L929 cell line in the MTT test, cultured with different concentrations of ZnO NWs for 12 h, 24 h, 48 h. Adapted with permission from reference [87]. Copyright (2008) American Chemical Society.

After seeding, HeLa cells started to grow and reproduce well even in the presence of the NWs, with some nanostructures being readily phagocytosed from the ingrowing cells (Figure 2). The biocompatibility of ZnO NWs was further assessed in vitro by MTT assay, where MTT is the name of the dye 3-(4,5-dimethylthiazol-2-yl)-2,5-diphenyltetrazolium bromide, reduced during the tests), monitoring the activity of the mitochondrial enzyme succinate dehydrogenase (SDH). Figure 3a shows that no appreciable effects on HeLa cell viability occurred after 12 and 24 h. A slight reduction was obtained only in the presence of the highest NW concentrations. In this case, more than 95% of cells were still viable after 24 h, while cell viability dropped to around 75% after 48 h. In contrast, the viability of L-929 cells was found to be time-dependent and affected by NW concentration (see Figure 3b). After culturing for 12 h, cell viability was generally lower than after 24 and 48 h, indicating that L-929 cells were more sensitive towards the exposition to ZnO NWs during the first hours. Moreover, after 48 h cell viability significantly dropped to around 50% in the presence of the highest NW concentrations (100 μg·mL^−1^).

**Figure 4:**
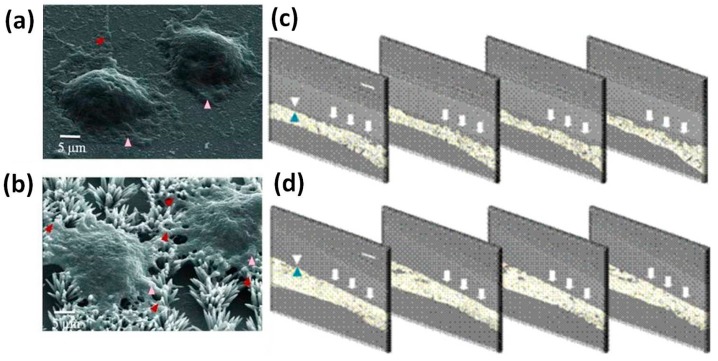
Panels (**a**,**b**): Scanning electron microscopic images of MC3T3-E1 osteoblasts grown on (**a**) ZnO film and (**b**) ZnO nanoflowers after cultivation for a day. Lamellipodia and filopodia were indicated with pink and red arrowheads, respectively. Panels (**c**,**d**): sequential synchrotron X-ray tomographic images of regenerated bones to (**c**) ZnO film and (**d**) ZnO nanoflowers on ZnO film-coated silicon substrate implanted in the calvarial bone defects of rats for four weeks. Arrows (▲ or ▼) indicate the interface between ZnO surface and the regenerated bone exhibiting the topographic effect of ZnO nanoflowers on osseointegration. Scale bars correspond to 100 μm. Modified with permission from reference [15]. Copyright (2010) John Wiley & Sons, Inc.

A more detailed study of the biocompatibility of one-dimensional ZnO nanostructures was carried out by Park et al. [15], which studied the growth and osseointegration of MC3T3-E1 osteoblast cells cultured on two completely different ZnO structures: NFs and flat thin films. The characteristics of the cultured cells were first evaluated in vitro, analyzing lamellipodia and filopodia formation, DNA content and ALP activity. After four days, ZnO NFs were completely covered by osteoblasts, as shown in panel a and b of Figure 4. The lamellipodia formation was obtained both on ZnO thin films and NFs, although NFs resulted in more active filopodia formation. This aspect was a first evidence of the topographic effect due to the higher specific surface area exposed from the NFs. This positively affected fibronectin adsorption, integrin binding and the expression of cytoskeletal protein, finally promoting more filopodia formation than ZnO thin film. Osteoblasts cultured on ZnO NFs also expressed significantly higher values for DNA content and ALP activity, respectively. To further demonstrate the topographic effect of ZnO NFs, the adhesion of osteoblasts was investigated after the application of a constant centrifugal force of 7.8 pN at 4 °C. The adhesion of cells was better in the case of ZnO NFs, with an adhesion strength 55.8% higher with respect to the ZnO thin film.

Finally, the osseointegration properties of ZnO NFs and thin films deposited on silicon (Si) substrates were investigated upon their implantation in vivo for four weeks into the calvarial bone defects of rats. Bone regeneration was obtained for both the ZnO structures. However, sequential synchrotron X-ray tomographic analyses showed that the regenerated bone tissue was poorly adhered to ZnO thin film (Figure 4c), while being more osseointegrated in the case of ZnO NFs. In such a situation, osteoblasts stuck more tightly and without any gap between the implanted device and the regenerated tissue, as clearly visible in panel d of Figure 4. 

The ability of ZnO nanomaterials to promote osteoblast growth was also considered by Foroutan et al. [88]. Here, ZnO NPs with an average size of 30 nm were dispersed in osteogenic medium at different concentrations (30 and 60 μg·mL^−1^). Then, bone marrow mesenchymal stem cells (MSCs) were cultured up to 15 days in the presence of the ZnO NPs and the effect on MSC differentiation into osteoblasts was evaluated. To this purpose, alizarin red staining and real-time polymerase chain reaction (RT-PCR) assays were carried out to quantify ALP activity and the expression of osteopontin and osteocalcin (OCN). After 15 days, both the analyses evidenced that MSCs successfully differentiated into osteoblast cells in the presence of the NPs, with higher levels of osteopontin, OCN and ALP expression for the group of cells treated with the lower dose of NPs, i.e., 30 μg·mL^−1^. Instead, high levels of toxicity were observed for the highest concentration, 60 μg·mL^−1^. Therefore, administering ZnO NPs in vitro at specific concentrations (30 μg·mL^−1^) and with a particular size (~30 nm) actively contributed to the osteogenic differentiation of MSCs into osteoblasts. 

The in vitro biocompatibility of ZnO was also evaluated by Ramesh and coworkers [89]. In this case, epitaxially-grown ZnO NRs were obtained by the hydrothermal method and then dispersed in PBS at different concentrations. The biocompatible properties of NRs were assessed by analyzing both their interaction with rat lung epithelial (LE) cells and their ability to generate reactive oxygen species (ROS), which are well-known markers for oxidative cellular stress. The alteration of oxidative stress levels was first examined by treating LE cells for 3 h with ZnO NR solutions at increasing concentrations, lying in the range 0.5–20 μg·mL^−1^. After the exposure, no increase in oxidative stress or lipid peroxidation in the exposed LE cells was observed, even at the highest time and concentration levels. The MTT assay further showed that cell viability was independent of ZnO NR concentration and time of exposure, as it was always higher than 95%. The absence of cytotoxicity, ROS generation and oxidative stress in LE cells was due to the low concentration of zinc ions (Zn^2+^). Indeed, Zn^2+^ is often related to cytotoxic phenomena and oxidative stress conditions. The existence of a threshold value in Zn^2+^ concentration was then supposed to exist. Such a threshold was never achieved during the experiments, thereby not negatively affecting ZnO NR biocompatibility and LE cell viability.

**Figure 5:**
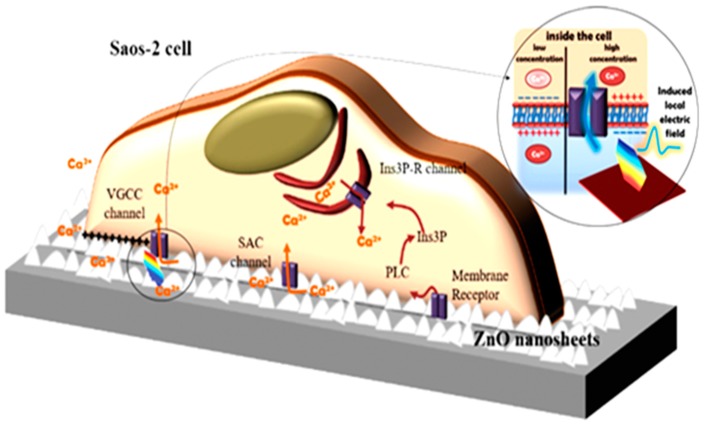
Sketch of a cell grown on top of a nanogenerator (NG) array indicating the possible pathways involved in changes of the cytosolic Ca^2+^ concentration. Extracellular Ca^2+^ influx is due to the opening of plasma membrane channels, either voltage gated Ca^2+^ channels (VGCC) or stretch-activated cation channels (SACC). By contrast, intracellular Ca^2+^ comes from endoplasmic reticulum stores through the activation of membrane receptors and the opening of Ins3P-receptor channels. The bending of a NG would induce a local electrical field proportional to the strain level that could eventually alter the membrane potential and/or the configuration of membrane receptors and results in the opening of the Ca^2+^ channels. Adapted with permission from reference [93]. Copyright (2017) John Wiley & Sons, Inc.

As previously mentioned, ZnO nanostructures have been widely investigated also for their promising piezoelectric properties [55,76,90]. The use of ZnO nanomaterials as nanogenerators (NGs) could thus be envisioned, finding widespread application in new generation energy harvesting systems and nanosensors [91,92]. Very recently, ZnO NGs have been successfully tested also for the electrical stimulation of cells. Living human cells (macrophages and osteoblast-like Saos-2 tumor cells) were cultured on ZnO nanosheets (NSs), and their mutual interaction was investigated [93]. The aim, sketched in Figure 5, was to demonstrate how the mechanical forces excercised from the living cells on the underlying piezoelectric ZnO nanosheet array could induce the rise of a local electric field, and how this local field affected the biological behavior of the grown cells.

**Figure 6:**
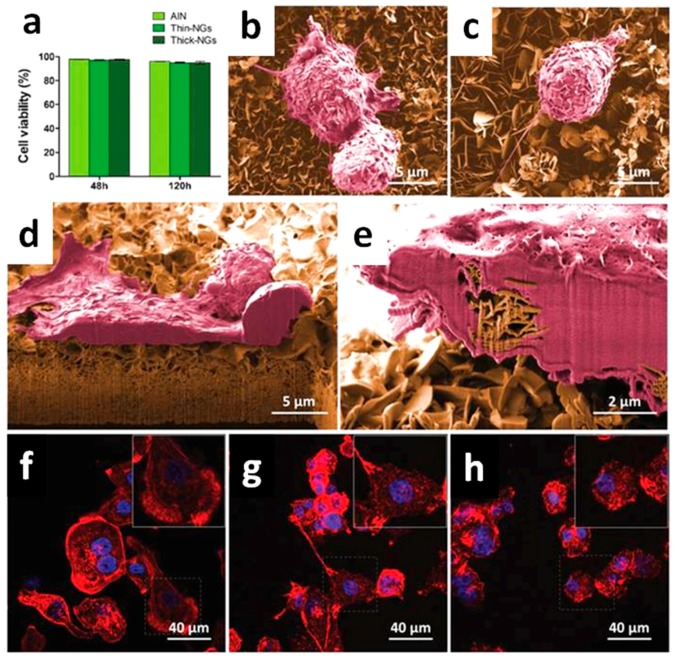
Biocompatibility of the thin- and thick-nanogenerators (NGs) and control aluminum nitride (AlN) for macrophages. (**a**) Cytotoxicity was analyzed by the live/dead kit at 2 and 5 days of culture. No differences were found among the samples and time-points analyzed (*χ*^2^ test); (**b**,**c**) Cell morphology and NG–cell interaction, assessed by scanning electron microscopy and focus ion beam after 2 days in culture, showed that macrophages adhered to both (**b**) thin and (**c**) thick NGs; (**d**,**e**) The plasma membrane was in direct contact with the (**d**) thick NGs and some macrophages even appeared to have engulfed the (**e**) thin NGs. (**f**–**h**) Confocal laser scanning microscopy images of actin filament (red) detection showed that the distribution of stress fibers was similar on cells grown on (**f**) AlN; (**g**) thin NGs; and (**h**) thick NGs (see the enlarged area). The nuclei are stained in blue with DAPI dye. Scanning electron micrographs were false-colored for better visibility, depicting macrophages (magenta) and nanogenerators (yellow). Modified with permission from reference [93]. Copyright (2017) John Wiley & Sons, Inc.

**Figure 7:**
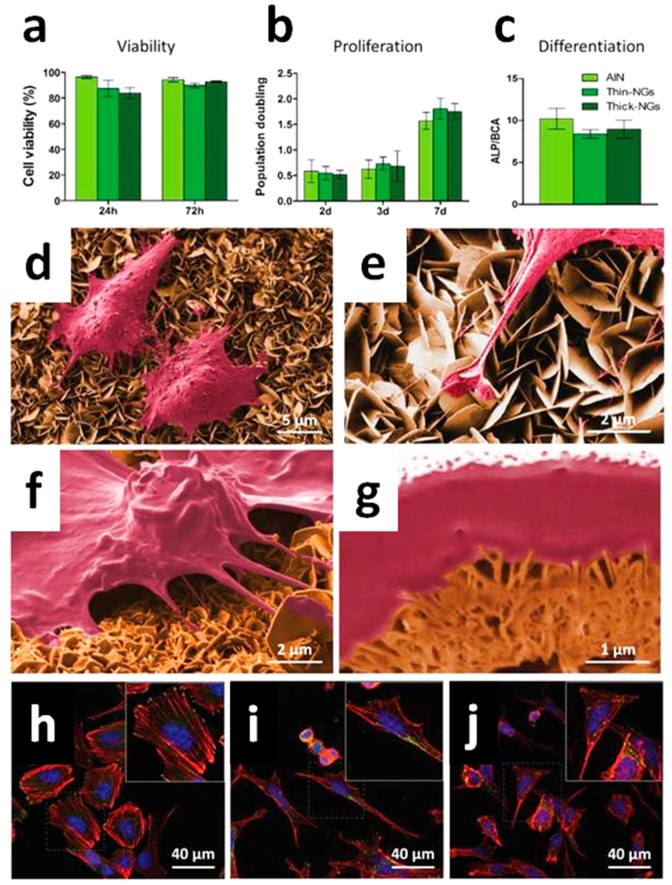
Biocompatibility of the thin and thick nanogenerators (NGs) and control aluminum nitride (AlN) for Saos-2 cells. (**a**) Viability was analyzed by the live/dead kit at 24 and 72 h of culture; (**b**) Proliferation by the Alamar Blue assay at 1, 2, 3, and 7 d; (**c**) Differentiation by a quantitative analysis of alkaline phosphatase activity (ALP) activity, a differentiation osteoblast marker, at 14 days of culture. No significant differences among materials were observed for any of those parameters (*χ*^2^ test and Kruskal–Wallis test); (**d**,**e**) Morphology and NG–cell interaction, assessed by scanning electron microscopy (SEM) and focused ion beam (FIB), showed that cells were firmly adhered to the nanosheets (NSs) (**d**) and emitted long projections that were anchored to the NSs (**e**); (**f**,**g**) Cells were completely adapted to the topography of the NSs (**f**), which were in direct contact with the plasma membrane (**g**); (**h**–**j**) Confocal laser scanning microscopy analysis after immunodetection of vinculin (focal contacts, green) and detection of actin (stress fibers, red) showed a high number of focal contacts at the end of long parallel bundles of actin filaments on cells grown on AlN, displaying a polygonal shape (**h**). The nuclei are stained in blue with DAPI dye. Shorter actin bundles and less focal contacts were present in cells grown on thin NGs (**i**) and thick NGs (**j**), which also displayed a more elongated, spindle-shape morphology (see the enlarged area). Scanning electron micrographs were false-colored for better visibility of the Saos-2 cells (magenta) and the nanogenerators (yellow). Modified with permission from reference [93]. Copyright (2017) John Wiley & Sons, Inc.

The modulation of cell responses, like macrophage motility and the opening of Ca^2+^ channels within the plasma membrane of osteblast-like cells with intracellular calcium transients, was achieved due to the ZnO NGs, without applying any external physical stimulus. These findings were possible due to the reduced thickness of the NS array (<20 nm) coupled to their high surface area, piezopotential generation properties, and the compatibility between piezopotential amplitudes generated by the NSs and the typical cell membrane potential. Once cultured atop the ZnO NSs, the inherent forces from the living cells (0.1–10 nN) allowed the bending of the NSs, creating a local electric field due to ZnO piezoelectric effect. The generated piezopotential (0.5–50 mV) resulted in the modulation of macrophage motility and Ca^2+^ channel opening. The biocompatibility of the two-dimensional ZnO NGs was first assessed both for macrophages (Figure 6) and osteoblast-like cells (Figure 7). Cell viability, adhesion, proliferation and differentiation were found to be not negatively influenced by the presence of the ZnO NSs. Both the cell lines adhered well to the ZnO NS array, showing the plasma membrane in close contact with the ZnO array (panels d of Figure 6 and Figure 7), the emission of short and long projections attached to single NSs (Figure 7e), and the presence of stress fibers and focal contacts (panels h of Figure 6 and Figure 7). 

**Figure 8:**
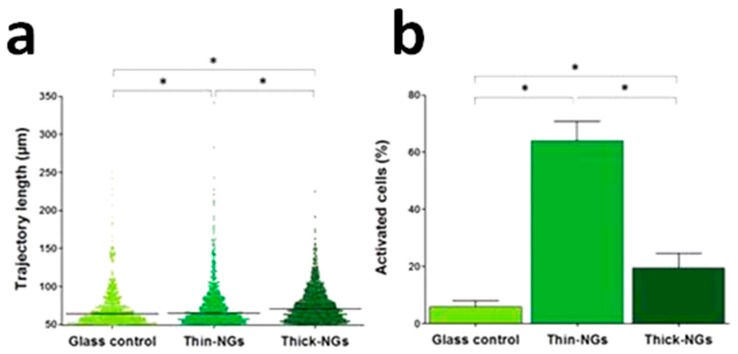
Effect of the thin- and thick-nanogenerators (NGs) on macrophages and Saos-2 cell activity. (**a**) Macrophage motility quantified as trajectory lengths. Distances shorter than 50 μm were considered in situ motions; (**b**) Quantification of Saos-2 cells undergoing changes in Ca^2+^ concentration (activated cells). Asterisks (*) above the columns indicate significant differences between data sets, according to Kruskal–Wallis test and *χ*^2^ test. Modified with permission from reference [93]. Copyright (2017) John Wiley & Sons, Inc.

Finally, the effect of the ZnO piezopotential on cellular activities like motility and intracellular Ca^2+^ concentration was investigated. Figure 8a shows that the macrophage motility was 36.7% higher than the control sample, with a travelled path longer than 50 μm. Moreover, the number of cells travelling for distances ranging from 100 to 150 μm triplicated. On the other hand, around 64% of Saos-2 cells cultured atop the two-dimensional ZnO NSs showed an increased Ca^2+^ concentration, with amplitudes of Ca^2+^ transients compatible with the influx of extracellular calcium, as visible from Figure 8b. To explain such achievements, the authors hypothesized that the piezopotential generation from the ZnO NSs due to the inherent forces from the cultured cells was high enough to trigger the opening of voltage-gated calcium channels (VGCC) or stretch-activated cation channels (SACC) of Saos-2 cells, allowing the influx of extracellular Ca^2+^ and producing high amplitudes of Ca^2+^ transients.

**Figure 9:**
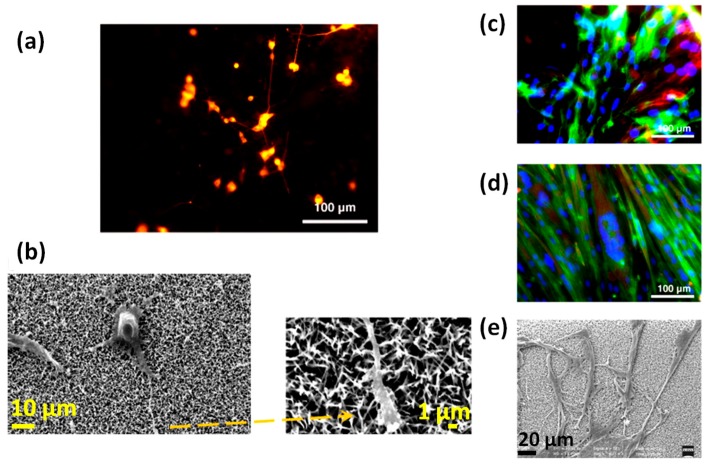
(**a**,**b**) PC12 differentiation: immunostaining of β3-tubulin (in red, (**a**)) and SEM imaging (**b**). Higher magnification of the SEM picture is shown as inset of panel (**b**), depicting that ZnO nanowires penetrated the cell membrane. (**c**–**e**) H9c2 differentiation: fluorescent staining of myosin heavy chain (in red), f-actin (in green) and nuclei (in blue) over ZnO nanowire arrays (**c**) and over standard polystyrene substrates (**d**); SEM imaging over ZnO nanowire arrays (**e**). Modified with permission from reference [13]. Copyright (2012) Elsevier.

Beyond osteoblast-like tumor cells, other primary cellular lines were cultured on ZnO nanostructures, and the resulting interaction was investigated. For example, Ciofani et al. [13] studied the adhesion, proliferation and differentiation of two different electrically-excitable mammalian cell lines on ZnO NWs array: PC12 line, modeling neuronal cells, and H9c2 line, as a model for muscle cells. Independently of the line, cells were highly viable (>95%) after culturing for 72 h, with lots of cellular protrusions and a conformal contact between cells and the ZnO NW array. To evaluate the differentiation behavior of the cultured cells, the immunofluorescece staining of specific markers (β3-tubulin for PC12 cells and myosin heavy chain, MHC, for H9c2 cells) was evaluated. After seven days from the differentiation induction, different behaviors were observed. Panels a and b of Figure 9 show that the PC12 cells exhibited a well-developed neurite network, with neurite approaching 100 μm in length. On the other hand, H9c2 cells displayed a disordered arrangement (Figure 9c), without showing the typical tubular shape of H9c2 myotubes, as visible in Figure 9e. Such a discrepancy in differentiation behavior was ascribed to the mechanical properties of the NW substrate. The authors claimed that neuronal cell adhesion and differentiation was prone to be obtained on the NWs because of their mechanical stiffness. On the other hand, the differentiation of muscle cells into myotubes requires a softer environment to be pursued. This is in contrast with the stiffness of the ZnO NW substrate, that is considered to be too rigid and hence not suitable for promoting myotube formation. Another factor that could influence differentiation is the nanotopography of the underlying substrate. Depending on the cultured cell, adhesion to the substrate is different and strongly affected by the particular nanotopography. Within this aim, the authors hypothesized that H9c2 adhered more tightly to ZnO NWs than neurites, hence preventing myotube formation.

Several studies also reported the promising use of nanostructured ZnO as an adhesion-resistant biomaterial. In this sense, the pioneering study of Lee et al. successfully demonstrated the possibility to control cell adhesion and viability on the surface of glass substrates coated by hydrothermally-grown ZnO NRs [14]. In this study, three different cell lines were compared: NIH 3T3 fibroblasts, human umbilical cord vein endothelial cells (HUVECs) and bovine capillary endothelial cells. The spreading of the cultured cells was first investigated in vitro, by evaluating focal adhesions and stress fibers. Compared to the flat ZnO substrate and the control sample, the adhesion and viability of fibroblasts and endothelial cells was strongly reduced on ZnO NRs. Even though cells were able to attach to the ZnO NRs, the absence of lamellipodia formation witnessed by scanning electron microscope (SEM) images evidenced their inability to spread on the NRs. This was also depicted by the decreased area of spreading cells (60–70% lower than flat ZnO and the control sample) and the round-shaped aspect of the few cells adhered to ZnO NRs, representing the loss in cell viability compared to flat ZnO. The lack of focal adhesion assembly in cells cultured on ZnO NRs was due to the excessive spacing between the ZnO NRs (~100 nm), while distances lesser than 70 nm should be present to promote focal adhesion assembly. 

**Figure 10:**
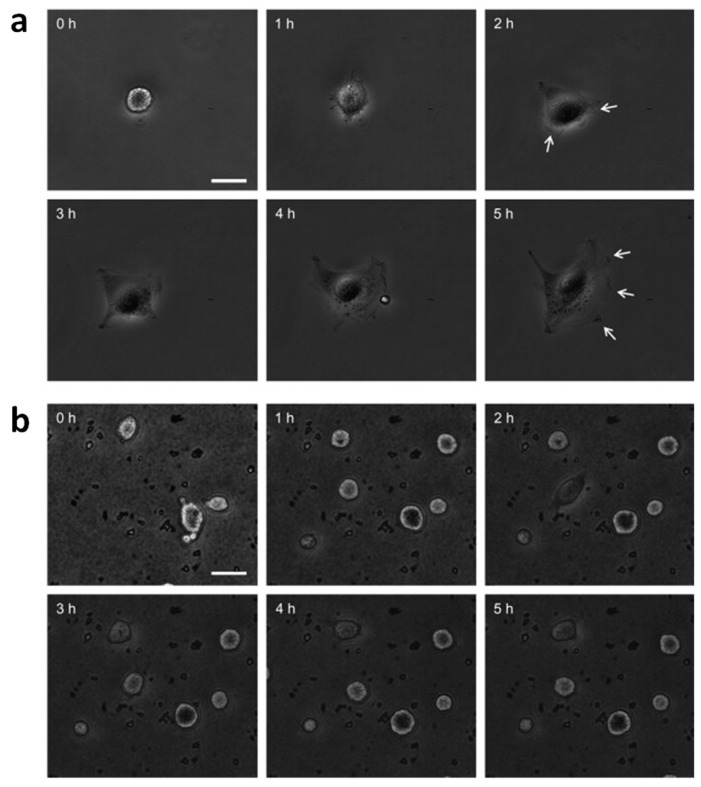
Dynamic cell spreading is altered by nanorods. Phase contrast imaging of human umbilical cord vein endothelial cells (HUVECs) spreading on glass and ZnO nanorods. (**a**) Cell spreading HUVECs is accompanied by lamellipodia formation (white arrows) and is complete in approximately 5 h; (**b**) Cells on nanorods do not spread, and do not develop any lamellipodia. Scale bar is 20 μm. Modified with permission from reference [14]. Copyright (2008) Elsevier.

The total number of adhered cells and of adhered-live cells after 24 h was generally reduced due to the presence of ZnO NRs. By evaluating the ratio between the number of cells attached to ZnO NRs over those attached to flat ZnO, the authors also stressed the effect of the substrate topography. Despite this ratio being similar for each cell type adhered to ZnO NRs and the flat sample, a significant difference in the number of living cells was observed. When considering ZnO NRs, the survival of endothelial and fibroblast cells decreased up to around 7% and 65%, respectively. As already mentioned, the engulfment of ZnO nanostructures from cultured living cells may occurr. To elucidate the eventual role played by NR engulfment on cellular apoptosis, time-lapse imaging was performed, to study dynamic cell spreading on ZnO NRs, and the results are collected in Figure 10. The initial adhesion of HUVECs occurred on the control glass sample during the first hour. On the other hand, lamellipodia formation could be seen from 2 h onward, followed by complete spreading at approximately 5 h. On the contrary, little initial spreading occurred on ZnO NRs, with round-shaped cells even after several hours. No lamellipodia formation was visible, providing evidence that NRs and their particular nanotopography did not support initial cell spreading. Another aspect that influenced cell behavior was the aspect ratio of NRs. The authors hypothesized that the engulfment of NRs by cells may have occurred due to the similarity between the aspect ratio of their NRs and those reported by other works, in which cellular apoptosis was observed due to the engulfment of nanostructures from the cultured cells. Therefore, the cytotoxic effects due to the engulfment of ZnO NRs could not be excluded, leading to the observed decrease in cellular survival.

**Figure 11:**
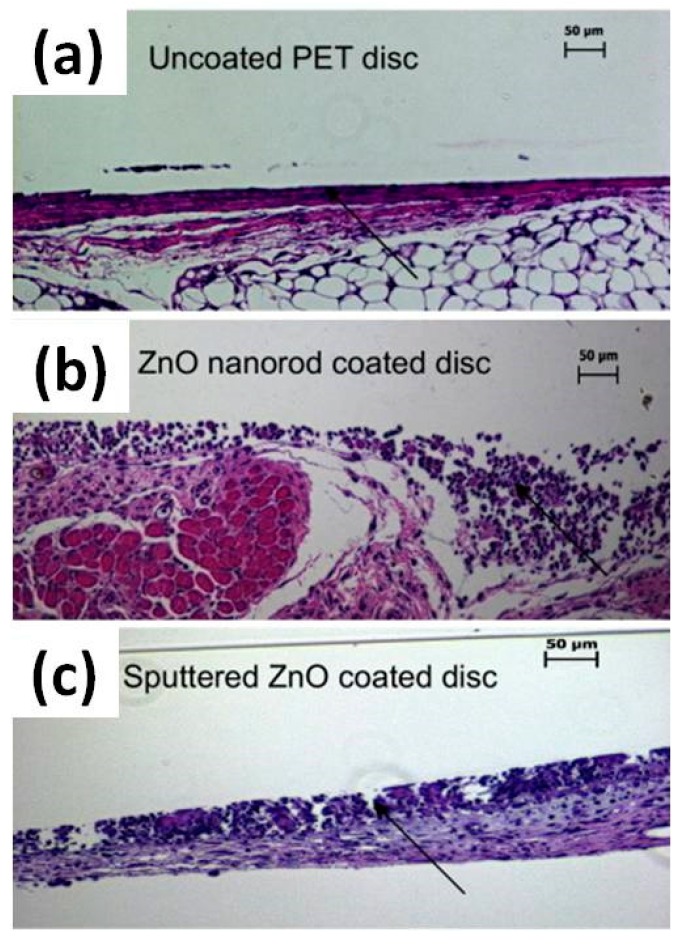
Zinc oxide coatings on implanted discs prevent formation of acellular fibrous capsules around discs, indicative of unresolved inflammation. (**a**) Verhoeff-van-Geisson stained section (collagen pink; cell nuclei: dark blue) of tissue response to implanted biomaterials. Uncoated polyethylene terephthalate (PET) disc has a smooth, relatively acellular fibrous capsule formed around the disc, indicated by the arrow; (**b**) PET discs coated with ZnO nanorods did not have an acellular fibrous capsule formed around discs; (**c**) PET discs coated with sputtered ZnO also did not have an acellular fibrous capsule formed around discs. On the contrary, the ZnO-coated discs demonstrate a lack of fibrous capsule formation and an accumulation of recruited inflammatory cells (indicated by arrows). Scale bar is 50 μm. Modified with permission from reference [94]. Copyright (2010) Elsevier.

The nanotopography and cytotoxicity of ZnO NRs and sputtered films on macrophages was further investigated by Zaveri et al. [94]. Bone marrow-derived macrophages were cultured on ZnO NRs. Despite initially adhering to and spreading over ZnO NRs, a retraction of lamellipodia appeared after 2 h together with the appearance of totally round-shaped macrophages after 3 h. Moreover, evident protrusions containing actin started to be present after 6.5 h, suggesting cytoplasmic leakage. Cell viability was also considered. After 16 h, the number of viable cells was significantly reduced compared to flat ZnO and the control sample, suggesting that nanotopography played a central role in determing cell viability. In particular, the total number of adherent living cells was 52% and 12% of the flat ZnO and control sample, respectively. The mechanism inducing cell death was also investigated. It was found that, independently of the surface nanostructure, i.e., NRs or flat ZnO thin film, most of the cells underwent necrosis, while only 1% were involved in apoptosis. This suggested that, beyond nanotopography, ZnO cytotoxicity played a key role in determining cell death. To further elucidate this last aspect, the same cells were cultured in the presence of leachates due to the dissolution of ZnO into Zn ions. After exposing the cells to a culture medium that was previously in contact with both the ZnO NRs and thin films, i.e., medium containing ZnO leachates, a decrease in cell viability of around 50% to that obtained for the control sample was observed after seven days. This demonstrated that both ZnO nanotopography and cytotoxicity played a key role in determining cell viability, modulating in this case macrophage adhesion and viability. In vivo analyses were also carried out. Subcutaneous implantation of polyethylene terephthalate (PET) discs coated with both sputtered ZnO thin film and NRs was performed for 14 days in mice. Histological analyses of the explanted discs are shown in Figure 11 and unveiled the formation of high levels of accumulated leukocytes together with the lack of a continuous fibrous capsule only in the case of ZnO-coated PET discs. These factors demonstrated the presence of unresolved inflammation and further confirmed the in vitro results of the study.

**Figure 12:**
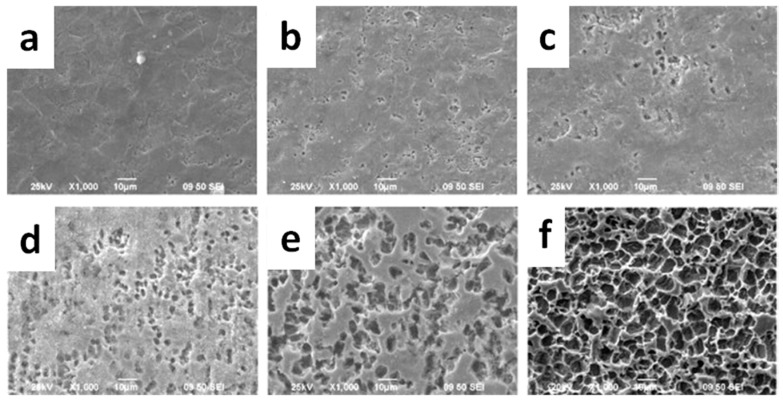
SEM images of zinc oxide films with different pore densities prepared by the anodic oxidation technique using different oxidation times. (**a**) 2 min; (**b**) 5 min; (**c**) 10 min; (**d**) 15 min; (**e**) 90 min; (**f**) 120 min. Modified with permission from reference [95]. Copyright (2011) Elsevier.

Prevention of macrophage adhesion and viability was also obtained by Wang et al., in the case of porous ZnO films [95]. This work mainly focused on demonstrating the pore-density-dependent cytotoxicity of ZnO films on the metabolism and spreading of fibroblasts. ZnO thin films with different porosities were fabricated by anodic oxidation of metallic Zn sheets with different oxidation times, as shown in Figure 12.

**Figure 13:**
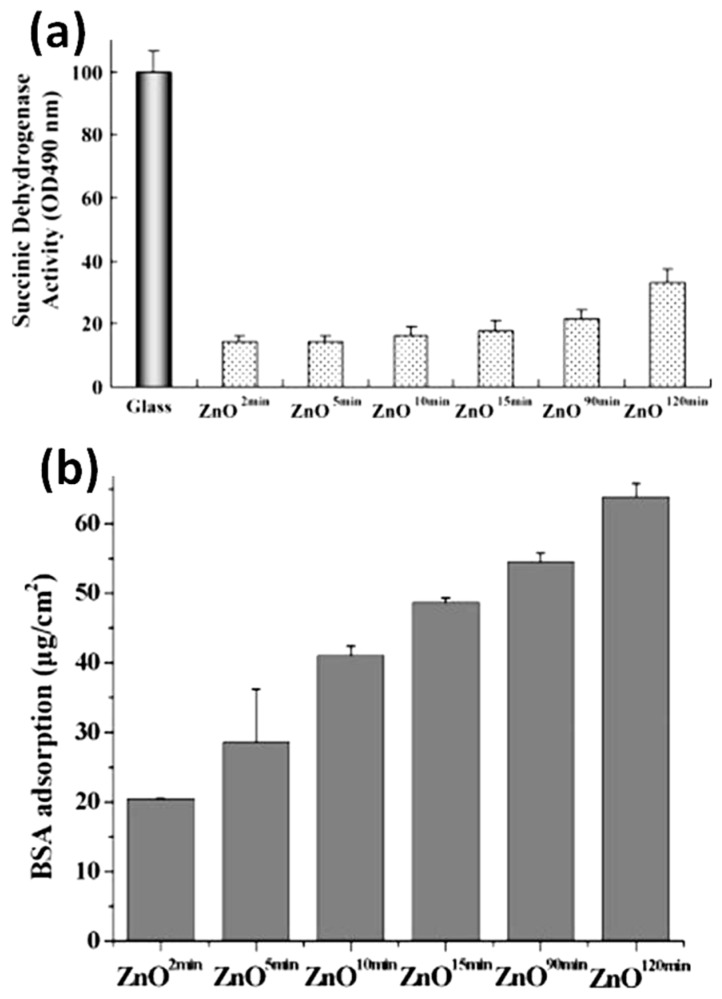
(**a**) Metabolic activity of NIH 3T3 cells cultured on ZnO films with different pore densities determined by the MTT method based on SDH activity. Cells were seeded at 10^4^ cells/cm^2^ and allowed to proliferate for 48 h. The data were expressed as mean ± SD (*n* = 3); (**b**) BSA adsorption on porous ZnO films with different pore densities in 30 min. Modified with permission from reference [95]. Copyright (2011) Elsevier.

NIH 3T3 fibroblasts were cultured for 48 h on porous ZnO films and their morphology and distribution evaluated by fluorescence microscopy. The obtained results showed that the proliferation and spreading of fiborblasts was significantly inhibited by the presence of the porous ZnO films. The round-shaped aspect of the cultured cells also suggested low metabolic activity. However, the inhibition of cell viability was found to be pore-size dependent. Despite being lower than the control group, cell viability improved by increasing the film porosity; SDH activity levels increased from around 14.4% to 33.4% when pore densities increased from 0.25% to 59.8%, as shown in panel a of Figure 13. This was due to the increased surface area of the most porous ZnO films, that were prone to supply more active sites for protein adsorption, hence promoting cell adhesion and stimulating the growth of the adhered fibroblasts. This hypothesis was confirmed by protein adsorption assay performed on the different porous ZnO samples. Actually, the bovine serum albumine (BSA) adsorption content increased with the increase of the ZnO pore density (panel b of Figure 13).

The work of Zaveri et al. ruled out the copresence of both nanotopography and cytotoxicity of ZnO leachates on the viability of macrophages cultured on ZnO NRs [94]. To further elucidate the role played by both these factors, Petrochenko et al. studied the cytotoxic effects of ZnO thin films grown by pulsed laser deposition and their leachates against macrophages [96]. In the first case, cells were cultured directly on ZnO-coated Si wafers. In the other case, ZnO-coated Si wafers were placed in medium for cell culture for 24 and 48 h. Then, leachates of those solutions were extracted and cells were exposed to such extracts. In order to evaluate a dose-dependent effect, the extracts were also diluted to get different ZnO leachate concentrations (1:1 and 1:3). Once cultured in direct contact for 24 h with the nanostructured ZnO surface, both MTT and flow cytometry assays confirmed a lower cell viability of 56.6% than the control sample. After 48 h, cell viability further decreased to 46.5%, with evidence of both nectrotic and apoptotic cell death. On the other hand, a stronger reduction in cell viability (up to 11.4%) was immediately observed when exposing macrophages to undiluted ZnO extracts for 24 h. No changes in viability and cell death were observed for the 25% and 50% extracts, indicating the existence of tolerable leachate concentrations. To better investigate the mechanism driving cell death, the generation of ROS was monitored in cells treated with ZnO extracts at various time points, over an exposure of 24 and 48 h. A remarkable increase in ROS production was observed only for cells treated both for 24 and 48 h with the undiluted extract. Therefore, it was concluded that in vitro tolerable amounts of ZnO (≤6.07 μg·mL^−1^) would exist, with highly-concentrated undiluted extract solutions (12–16.87 μg·mL^−1^) being considerably cytotoxic against macrophages.

**Figure 14:**
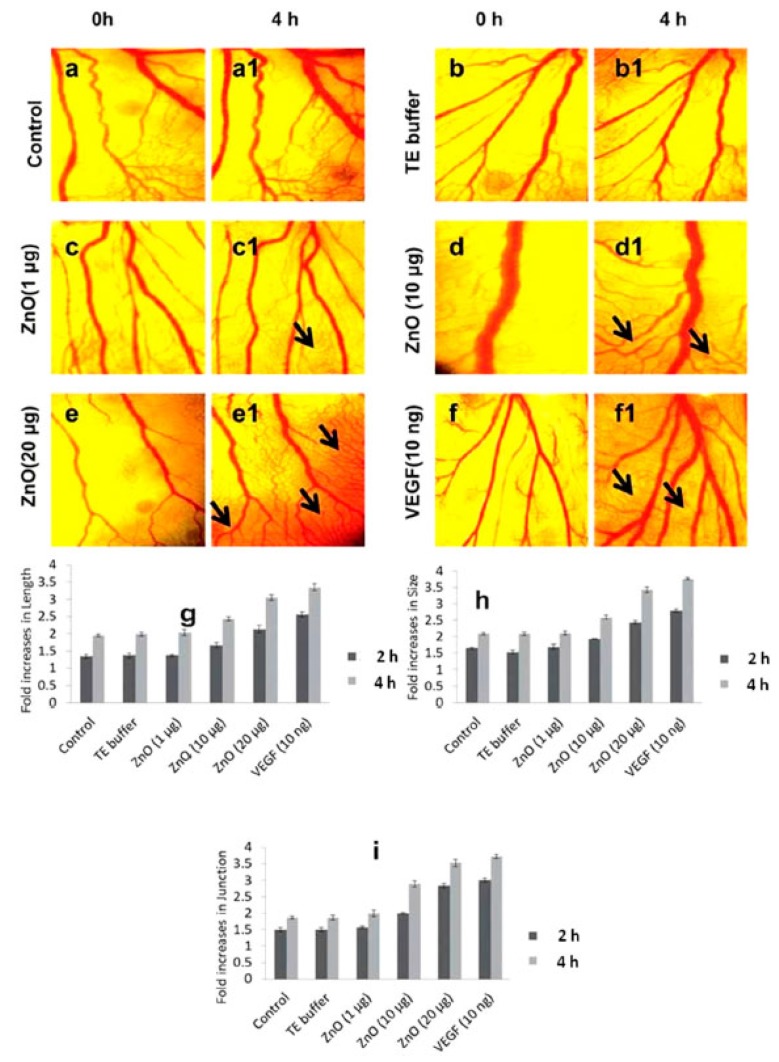
Chick embryo angiogenesis (CEA) assay with ZnO nanoflowers for in vivo angiogenesis assay. (**a**,**a1**) Untreated chicken egg yolks were considered as control; (**b**,**b1**) TE (Tris–Ethylenediamine Tetraacetic Acid) buffer; ZnO nanoflowers at different concentrations (1–20 mg) (**c**,**c1**,**d**,**d1**,**e**,**e1**); and vascular endothelial growth factor (VEGF) (10 ng) as positive control experiment (**f**,**f1**), respectively. A dose-dependent increase of matured blood vessel formation was observed with the increasing concentration of ZnO nanoflowers. The black arrows indicate the formation of new vasculature. Three angiogenesis parameters were quantified: vessel length (**g**); vessel size (**h**) and junction (**i**). Reproduced with permission from reference [20]. Copyright (2012) Royal Society of Chemistry.

Even though generation of ROS is often associated with cytotoxicity and necrotic cell death, Barui et al. successfully demonstrated for the first time the proangiogenic properties of ROS generated by ZnO NFs, and their use in wound healing applications [20]. ZnO NFs were suspended in a sterile buffer solution with a concentration of 10 mg·mL^−1^. The proangiogenic properties of the ZnO NFs were first evaluated by cell proliferation and cell cycle analyses. This last is of particular importance in the evaluation of the proangiogenic properties, since it represents the chain of events taking place in the cells leading to their division and replication/duplication. For these purposes, HUVECs were treated with ZnO NFs at different concentrations (0, 5, 10 and 20 μg·mL^−1^). Then, radioactive [^3^H]-thymidine and propidium iodide were fixed to evaluate cell proliferation and cycles. It was observed that ZnO NFs promoted HUVEC proliferation with respect to the untreated NFs, slightly indipendently of the ZnO dose. On the other hand, cell-cycle analyses highlighted that a significant percentage of the HUVECs were in the S-phase after administering ZnO NFs, i.e., the phase responsible for the synthesis of DNA and centrosomes. Finally, the proangiogenic properties were evaluated in vivo by a standard chick embryo angiogenesis (CEA) assay. Both untreated and egg yolks treated with ZnO NFs at different concentrations ranging from 1 to 20 μg were analyzed. The formation of new blood vessels took place in a dose-dependent way after 4 h, as visible in Figure 14. Additionally, ZnO NFs also promoted endothelial cell migration, showing the ability to close a scratch in a wound healing assay compared to the control sample. Both in the case of CEA and the wound healing assays, a green fluorescence emission signal due to the presence of H_2_O_2_ was detected only for HUVECs treated with ZnO NFs. H_2_O_2_ is a signaling molecule for angiogenesis which can be produced by mitochondria. Therefore, the authors hypothesized the oxidant role played by ZnO NFs, directly inactivating protein tyrosine phosphatase and tensin homolog in the cytoplasm via the oxidation of cysteine, finally leading to mitochondrial H_2_O_2_ production. Therefore, it was claimed that ROS generation could be a plausible mechanism for the observed angiogenic properties of ZnO NFs.

## 4. ZnO-Based Composite Materials

### 4.1. Bioactivity and Bone Tissue Regeneration Properties

New bone tissue formation requires the use of biomaterials able to promote the fast adhesion and proliferation of bone cells, coupled to a quick bioactive response. This means that scaffolds must be able to promote not only cell activity, but also the formation of hydroxyapatite (HAp), i.e., the mineral phase of bone, in a very short time. For these purposes, bioglass matrices have been widely investigated for bone TE, due to their well-known biocompatible properties coupled with a very fast bioactive response. However, such systems often suffer from fast degradation rates and poor mechanical properties. Therefore, the incorporation of ZnO nanostructures has been investigated as an alternative tool to overcome such limitations. 

Concerning the bioactivity properties of bioglass–ZnO composite systems, Kamitakahara et al. studied the bioactive behavior of CaO–SiO_2_–P_2_O_5_–CaF_2_ glass-ceramics, after the addition of ZnO powders (0–14.2 mol %) to partially replace CaO [97]. After soaking the samples in simulated body fluid (SBF), apatite formation was somehow suppressed. After seven days, low apatite formation was observed only for the sample incorporating the lowest amount of ZnO (0.7 mol %). On the other hand, no apatite formation was observed for ZnO amounts higher than 3.6 mol %. This loss in bioactivity was due to the increased chemical durability of the CaO–SiO_2_–P_2_O_5_–CaF_2_ system after incorporating ZnO. In particular, the presence of ZnO suppressed the formation of silanol groups on the bioceramic surface, in turn preventing the reaction between the bioceramic and the SBF solution to start. This was due to the very low solubility of ZnO in SBF and to its amphoteric behavior. The addition of ZnO (1, 3 and 5 wt %) to SiO_2_–CaO–P_2_O_5_ bioactive glass NPs was also considered by Ali and coworkers [98]. In this case, the presence of ZnO did not negatively affect the bioactivity, as all the glass powders exhibited the formation of an apatite layer on their surfaces after immersion in SBF. Similarly, Bini et al. investigated the bioactive behavior of SiO_2_–CaO–P_2_O_5_ glass powders, with and without ZnO [99]. The addition of ZnO at the expense of CaO and P_2_O_5_ was found to improve both the formation rate and final content of carbonated hydroxypatite (HCA) after soaking the samples in SBF for eight days. 

In order to improve the mechanical and degradation properties of bioactive CaO-SiO_2_ systems, Li et al. investigated the effect of partially substituting CaO with ZnO [100]. The considered bioceramics were prepared by sintering a sol-gel derived precursor, including zinc nitrate hexahydrate to substitute 5 mol % of ZnO for CaO. The morphological characterization pointed out that the addition of ZnO lowered the total pore volume, hence increasing the density of the ZnO-doped bioceramics. This directly resulted in an improvement of the bending strength and mechanical toughness. Moreover, the degradation behavior was tested after soaking the samples in Tris-HCl solution. After seven days, the weight loss of the ZnO-doped samples was significantly lower than the undoped sample. This confirmed the ability of the substituted ZnO to increase the density of the bioceramic, preventing the easy attack of the composite matrix by the Tris-HCl solution, finally leading to a lower degradation rate. Soaking the samples in SBF also confirmed that ZnO substitution did not affect the in vitro bioactivity properties, since the formation of a bone-like apatite layer was observed after seven days.

On the other hand, other commonly-used biopolymers like polycaprolactone (PCL) are known for their low degradation rates. To this purpose, the study proposed by Augustine et al. successfully showed that acceleration in PCL degradation may be obtained by incorporating ZnO NPs [101]. PCL nanofibers were prepared by the electrospinning method, without ZnO or incorporating different amounts of ZnO NPs (0.5–6 wt %). The degradation of the nanofibers was investigated in vitro, by soaking the samples in SBF for up to 30 days. A change in the crystalline structure of the PCL/ZnO composite fibers was observed after degradation experiments, together with a change in surface wettability due to the formation of functional OH and COOH groups. Both of these aspects indicated that hydrolytic degradation had occurred during the test. Moreover, the mechanical properties of nanofibers were reduced in the presence of ZnO NPs, especially at high ZnO loading. The faster degradation rate observed for the composite PCL/ZnO fibers was ascribed to the decreased crystallinity of the composite, but more interestingly also to the ability of ZnO NPs to produce ROS. Indeed, these species actively participated in the hydrolysis of PCL and promoted its faster degradation in the aqueous environment. 

The role played by ZnO NPs on the bioactive properties of PCL composite foams was also investigated [102]. No HAp precipitation was obtained after soaking PCL/ZnO foams in SBF solution for 15 days. However, the formation of a mineral phase was visible after 30 days. In general, HAp precipitation appeared mostly in the ZnO-doped samples, assuming the form of small dots rather than of a continuous layer. However, the presence of HAp became lower when increasing the amount of incorporated ZnO. The formation of HAp was mainly due to the presence of the OH group characteristics of ZnO NPs, coupled with the creation of additional functional groups deriving from the hydroxylation of PCL during the interaction with the SBF solution.

**Figure 15:**
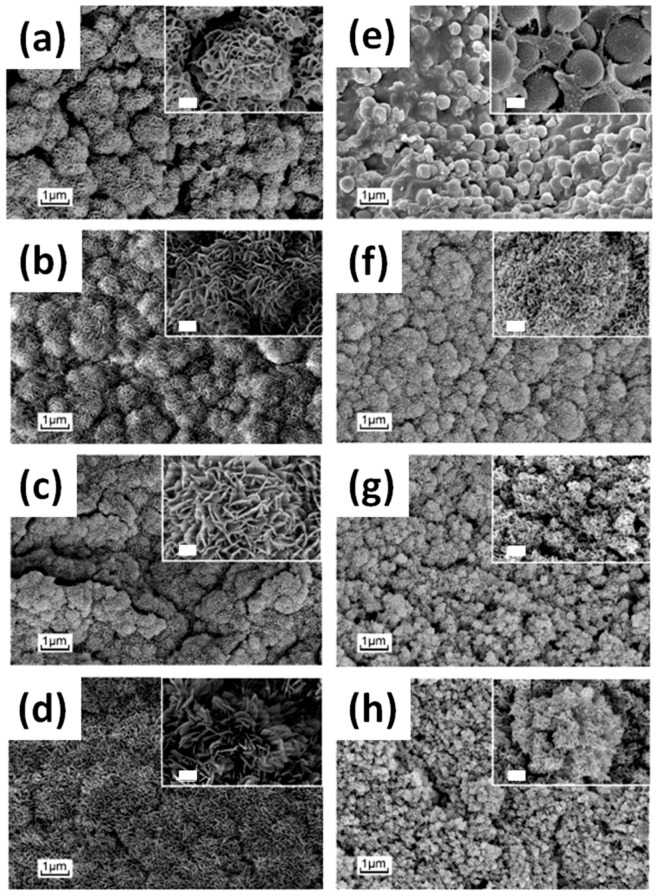
SEM images of the scaffolds after soaking in SBF, undoped scaffold: (**a**) 1 day; (**b**) 3 days; (**c**) 5 days; (**d**) 7 days; ZnO-doped scaffold: (**e**) 1 day; (**f**) 3 days; (**g**) 5 days; (**h**) 7 days. The insets are higher magnification (scale bar indicates 200 nm). Modified with permission from reference [103]. Copyright (2017) Elsevier.

More recently, the presence of ZnO nanostructures and their role in the apatite-forming ability of bioactive materials was also addressed by Guo et al. [103]. In this work, the bioactivity of glass/gelatine composite scaffolds reinforced with tetrapod ZnO whiskers in different amounts was investigated. Despite showing apatite layer formation, the release of Zn ions delayed the growth of HCA. Figure 15 shows the process of HCA formation as a function of the soaking time, both for the undoped composite scaffold (panels a–d) and the doped scaffold (panels e–h). When incorporating ZnO, HCA appeared as an amorphous and worm-like layer (Figure 15h) instead of crystalline and sponge-like. By considering the standard reaction model for HAp precipitation on bioactive glass systems, the abovementioned aspects were ascribed to multiple effects: (i) adding ZnO delays the release of Si ions from the bioglass system and promotes the formation of an CaO–P_2_O_5_ barrier layer, preventing HCA precipitation; (ii) the precipitation of phosphates is promoted; (iii) released Zn may substitute Ca in hydroxyapatite, leading to poorer crystallinity and crystal size.

Similarly to the SiO_2_–CaO–P_2_O_5_ systems, tricalcium phosphate (TCP) ceramics represent a milestone in the fabrication of scaffolds for bone TE, despite suffering from poor mechanical properties and fast degradation, which still prevent their use for load-bearing applications like orthopedic implants. In order to overcome such drawbacks, Feng et al. [17] successfully investigated the possibility to improve the mechanical and degradation properties of β-TCP scaffolds fabricated by a selective laser sintering technique, via the incorporation of ZnO NPs in different amounts (from 0 to 3.5 wt %). The biocompatibility of the composite scaffolds was investigated by analyzing the attachment and proliferation of osteoblast-like MG-63 cells. The mechanical properties (fracture toughness and compressive strength) and bioactivity of the resulting β-TCP-ZnO composite scaffolds were highly improved for an optimal amount of ZnO NPs (2.5 wt %). Once cultured on the surface of the composite scaffolds, osteoblast-like human cells were able to attach and proliferate better than for the control sample, i.e., β-TCP scaffold without incorporating ZnO NPs. However, it is worth mentioning that lower cell density and adhesion were found in the case of the highest incorporation of ZnO, i.e., 3.5 wt %. Finally, the formation of a bone-like apatite layer was successfully obtained after incubation of the optimized composite scaffolds (ZnO NPs 2.5 wt %) in SBF for 28 days. Such promising results suggested that Zn ions released from the β-TCP-ZnO composite scaffold actively contributed to the observed osteoinductive (formation of new bone tissue) and osteoconductive (guiding bone growth) properties, mainly because of the well-known property of Zn^2+^ to stimulate bone formation and mineralization [104], as well as its active role on the proliferation of osteoblastic cells [105]. Similar findings were reported also by Fielding et al. [19], who fabricated three dimensionally-printed TCP scaffolds incorporating 0.25 wt % ZnO NPs. The mechanical properties of the composite scaffolds were significantly improved, with a 2.5-fold increase in the mechanical compressive stress than for the pure TCP scaffolds. More interestingly, the biocompatible properties were also investigated in vitro, by seeding human fetal osteoblast (hFOB) cells. MTT assay showed that the cell proliferation rate was higher in the composite scaffolds with respect to the control sample, i.e., pure TCP. From SEM analyses a similar cellular morphology was obtained after the earliest culture days between the pure and doped TCP scaffolds. However, after seven days the formation of a complex filopodial communication network between neighboring cells was noticed in the doped TCP scaffold, which also showed an increased amount of living cells compared to the control sample. Also in this work, the osteoinductive properties due to the incorporation of ZnO within the TCP scaffolds were ascribed to the abovementioned stimulatory effects of Zn ions towards osteoblast cells. These findings were further demonstrated in vivo in a following publication from the same research group [18]. Specifically, β-TCP scaffolds were doped with ZnO NPs (0.25 wt %) and implanted in bicortical femur defects of a murine model for up to 16 weeks. After six and eight weeks from implantation, new bone formation was observed in the ZnO-doped scaffold, while after 12 weeks the scaffolds were completely infiltrated by the formation of new bone tissue. This was accomplished together with a significant increase in the formation of collagen type I at week six. OCN production and osteoclast activity were also monitored. Also in this case, the ZnO-doped samples showed increased levels of OCN at weeks six, eight and 12 when compared to the pure TCP scaffold. On the other hand, the osteoclast activity was comparable between the pure and doped TCP scaffolds throughout the overall experimental time. Finally, the authors also evaluated the degradation affecting the scaffolds, finding out that the doped TCP scaffolds better preserved their structural characteristics against the implantation time. The osteogenic properties of ZnO-doped TCP scaffolds described above were ascribed to the efficient release of Zn^2+^ ions from the doped samples, which provided an increased rate of bone regeneration, collagen type I formation, and OCN production.

Vallet-Regí and coworkers further investigated the osteogenic properties of Zn^2+^ ions by fabricating SiO_2_–CaO–P_2_O_5_ macro-mesoporous glass scaffolds incorporating ZnO [106]. The ZnO-enriched glass scaffolds were prepared by a 3D rapid prototyping method and their interaction with human osteoblast-like cells (HOS cells) was evaluated in vitro. After culturing for two days, HOS cells were in close contact with the scaffold surface, being viable and well-spread independently of the ZnO amount. Mitochondrial activity analyses carried out after one, three and six days revealed that cells proliferation improved by increasing the incubation time, except for the scaffold with the highest amount of ZnO. In this situation, adequate cell proliferation was observed only for the first three days. This was due to cytotoxic effects that affected cell proliferation, as confirmed by Lactate DeHydrogenase (LDH) assay. To evaluate the effect of ZnO on osteoblast differentiation, ALP activity was also monitored. Again, promising results were obtained after incubation for seven and 21 days; ALP levels significantly increased, reaching the maximum levels after 21 days and being three times higher than after seven days. However, the glass scaffold incorporating the highest amount of ZnO negatively affected osteoblast differentiation, inducing cytotoxic effects as mentioned above. Finally, the authors also studied the biocompatibility of the degradation products from the pure and ZnO-doped SiO_2_–CaO–P_2_O_5_ glass matrices. The scaffolds were soaked in medium for cell culture and the same medium was collected after two and four days, filtered and re-used for seeding HOS cells. LDH assay and the analysis of cell morphology indicated that no cytotoxic agent was generally released from the scaffolds, as osteoblasts maintained their typical shape. However, degradation products coming from the scaffold with the highest ZnO content (7 mol %) resulted in a partial agglomeration of osteoblasts; after four days, cells were able to adhere, but without spreading. 

**Figure 16:**
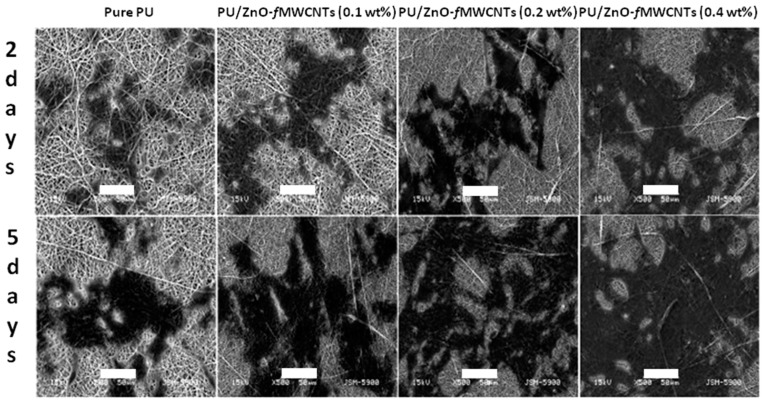
SEM micrographs showing adhesion and proliferation of MC3T3-E1 pre-osteoblasts on different nanofibrous scaffolds for two and five days. Scale bar is 50 μm. Modified with permission from reference [107]. Copyright (2017) Elsevier.

The incorporation of ZnO NPs, coupled with the presence of multiwall carbon nanotubes (MWCNTs), was found to effectively promote the osteogenic differentiation of pre-osteoblast MC3T3-E1, also in the case of polyurethane (PU) nanofibers [107]. After two, five and seven days of cell culture, the proliferation of cells was found to be 20.14%, 56.89% and 84.76% higher on PU–ZnO NPs-MWCNTs 0.4 wt % nanofibers than on the control PU sample. The SEM analyses shown in Figure 16 evidenced that pre-osteoblast cells spread more on the doped PU scaffolds, where a continuous increase in cell proliferation, differentiation and migration was obtained. Differentiation into osteoblasts was further confirmed by evaluating ALP activity and the secretion of collagen type I; in both the cases, the levels remarkably increased at seven and 14 days in the case of the hybrid PU nanofibers. Finally, the hybrid PU nanofibers significantly improved cell viability compared to pure PU nanofibers.

Bhowmick et al., also proved the biocompatibility of hybrid chitosan-poly(ethylene glicol)-nanohydroxyapatite (nHAp)-ZnO composite scaffolds and their ability to support the growth and proliferation of osteoblast-like MG-63 cells [108]. This was first due to the increased surface area of the nanocomposite, after incorporating ZnO NPs and nHAp; indeed, BSA results confirmed that protein adsorption was higher on the hybrid nanocomposite scaffolds than on the control sample, suggesting a better cell–matrix interaction, adhesion and spreading on the scaffolds. The MTT assay also evidenced that cell viability was higher on the hybrid system, but this was mainly due to the presence of nHAp. The in vitro proliferation study also confirmed that the hybrid scaffolds did not negatively affect the growth of osteoblast-like cells, therefore suggesting their potential use in bone tissue engineering. 

Very recently, the osteogenic activity of ZnO NPs/carboxylated graphene oxide nanocomposites for bone tissue engineering was successfully demonstrated [109]. Human osteosarcoma MG-63 cells were cultured in vitro on the nanocomposite scaffolds and the effect of releasing Zn^2+^ ions on cell viability and ALP activity was evaluated. The presence of ZnO NPs anchored to carboxylated graphene oxide allowed for a better control of Zn^2+^ release over time, avoiding the arise of deleterious cytotoxic effects against the cultured cells. However, the linkage between ZnO NPs and graphene oxide did not prevent the promotion of ALP activity and OCN secretion due to the Zn element. Indeed, after 14 days the levels of ALP and OCN were enhanced with respect to the control sample, i.e., not incorporating ZnO. Both the strict control over the release of Zn ions over time, coupled with its osteogenic properties, finally resulted in the enhancement of MG63 cell adhesion, viability and differentiation into osteoblasts, also promoting extracellular matrix mineralization.

### 4.2. Antibacterial Properties

The properties of ZnO as an antimicrobial agent have been often reported [16]. For example, Colon et al., investigated the antimicrobial properties of microphase and nanophase ZnO ceramic compacts [86], observing a decreased *Staphylococcus epidermidis* adhesion on nanophase ZnO. In particular, normalizing to the projected surface area, the *S. epidermidis* density and the corresponding formation of colony units on nanophase ZnO was 60% lower than on microphase ZnO. Such promising antibacterial effects were due to the synergic effect of releasing Zn ions coupled with the higher specific surface area of nanophase morphology, actually making for easier ZnO dissolution and ion release than for microphase ZnO. In order to confer antimicrobial properties, ZnO nanostructures were incorporated into both ceramic and polymeric matrices as well, resulting in hybrid ZnO-based composite materials with improved antimicrobial activities. 

**Figure 17:**
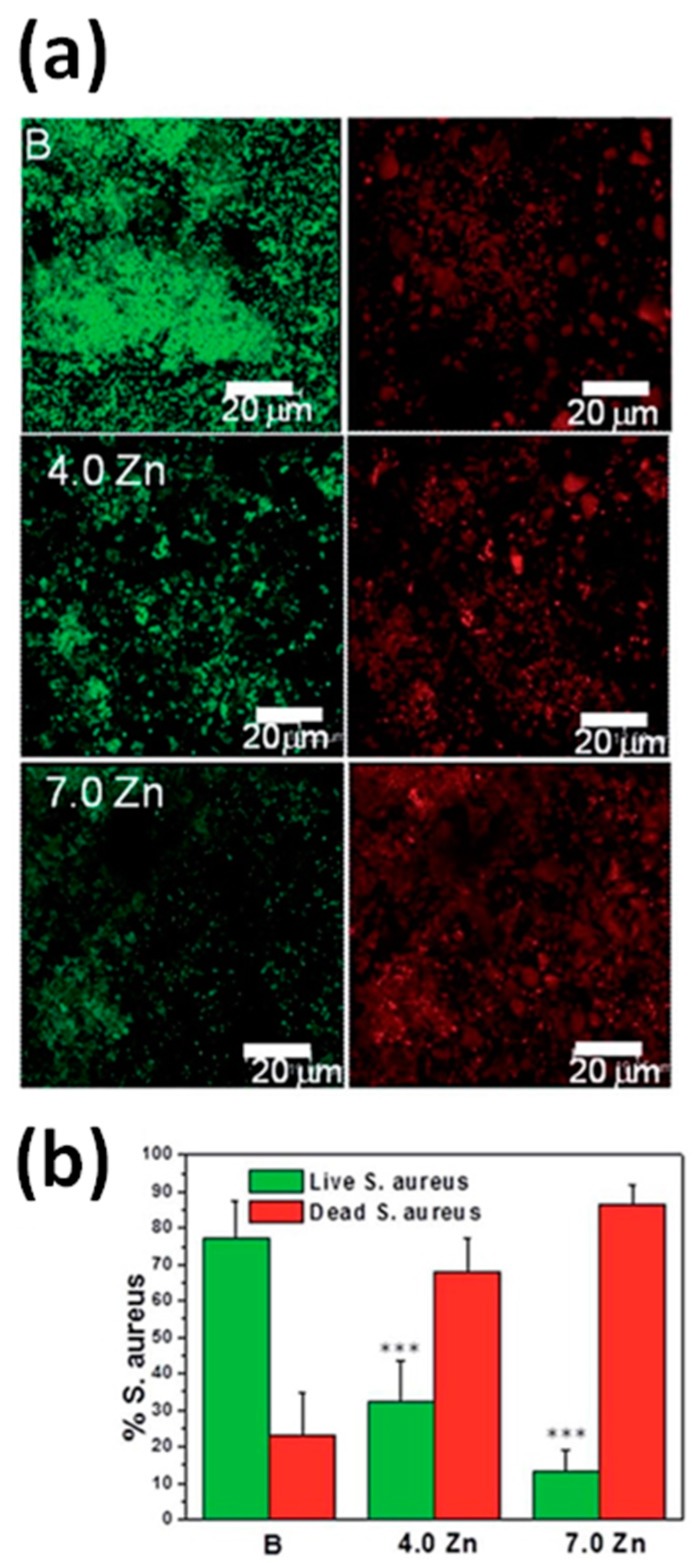
(**a**) Representative confocal micrographs of *S. aureus* biofilms formed on mesoporous glass scaffold surfaces after being soaked for two days in Todd-Hewitt Broth (THB) medium. Viable (green) and non-viable (red) bacteria are shown; (**b**) The percentages of the surface area covered with dead and live bacteria were calculated using ImageJ software (National Institute of Health Bethesda, MD, USA). The assay values shown are mean ± standard error. *** Significantly different compared to bare (B) scaffolds (*p* < 0.005). Reproduced with permission from reference [106]. Copyright (2014) Royal Society of Chemistry.

For example, the antimicrobial behavior of ZnO was exploited by Vallet-Regí and coworkers in ZnO-enriched SiO_2_–CaO–P_2_O_5_ bioactive glass scaffolds [106]. *Staphilococcus aureus* was considered a Gram-positive bacteria, responsible for infections in orthopedic surgery. The number of bacteria still viable after interaction with ZnO-doped glass scaffolds for two days was significantly lower with respect to the undoped control sample, as clearly visible from Figure 17. Moreover, bacteria viability strongly decreased (from 30% to 10%) by increasing the amount of ZnO within the glass scaffold from 4 to 7 mol %. These results were a clear indication that the incorporation of ZnO may lead to efficient antimicrobial properties for macro-mesoporous glass scaffolds. In particular, both ZnO incorporation and Zn^2+^ ion release prevented biofilm formation and effectively suppressed bacteria viability even for low concentrations of released Zn ions.

Very recently, the antibacterial properties of ZnO were exploited also in the case of ZnO/carboxylated graphene oxide (GO–COOH) nanocomposites, against *Streptococcus mutans* [109]. After seeding on the composites, the bacteria underwent a remarkable change in morphology, assuming a globular shape and showing decomposition of the membrane. The formation of inhibition zones was highly visible around both the GO–COOH and ZnO/GO–COOH nanocomposite samples. However, their extension was significantly higher in the case of ZnO GO–COOH. The observed antibacterial effects were due the co-presence of graphene oxide (GO) nanosheets and ZnO NPs. In particular, the authors assumed that the sharp edges of the GO nanosheets coupled with the abrasiveness of ZnO NPs physically damaged the bacterial membrane. On the other hand, the presence of a high local concentration of Zn^2+^ ions released from the composite also favored bacterial death.

Bottino and coworkers successfully incorporated ZnO NPs into PCL and PCL/gelatin polymer nanofibers deposited by electrospinning [110]. In order to show their potential application for periodontal regeneration, the antimicrobial properties of the developed hybrid composites were investigated against *Porphyromonas gingivalis* (*Pg*) and *Fusobacterium nucleatum* (*Fn*), i.e., well-known periodontal pathogens. All the investigated composite membranes generally showed good antimicrobial properties against both bacteria. However, the authors observed some differences in bacteria inhibition, dependent on the considered bacteria and amount of ZnO NPs. Higher inhibition zones (up to 11–14 mm) were obtained when using the PCL matrix with the highest amount of ZnO NPs (30 wt %). On the other hand, the presence of gelatin within the PCL matrix influenced the antimicrobial activity against *Pg*. In this situation, the dose-dependent effect was not so pronounced, with *Pg* bacteria being equally inhibited independent of the amount of ZnO NPs. Instead, the inhibition activity against *Fn* was found to be the best, with the inhibition zones being more extended than for *Pg*.

The antimicrobial activity of ZnO NP-filled electrospun PCL fibers were investigated as well [111]. Both Gram-positive *Staphylococcus aureus* and Gram-negative *Escherichia coli* bacteria were considered in the study. PCL fibers without incorporating ZnO NPs were used as a control. It was pointed out that a minimum amount of ZnO must be incorporated within the polymer in order for antimicrobial activity to be observed. Indeed, bacteria inhibition was evident only with the 5 and 6 wt % of ZnO NPs, while no inhibition action was observed at lower amounts of ZnO. This was due to the reduced presence of ZnO NPs, coupled to their being trapped inside the polymer fibers, which prevents their outer diffusion and contact with bacteria. The antimicrobial properties of the composite fibers against bacteria were also different. Inhibition of *S. aureus* was higher than *E. coli*. This discrepancy was due to their different cell wall structures, which influenced the interaction with antimicrobial ZnO NPs and their antibacterial efficacy. 

Similarly, Shalumon et al. incorporated ZnO NPs into sodium alginate/poly(vinyl alcohol) electrospun fibers and investigated their antimicrobial properties against *S. aureus* and *E. coli* [112]. After the incorporation of ZnO, the composite fibers showed inhibition activity against both bacteria, with *E. coli* being less inhibited by the ZnO NPs. Again, the inhibition zone for both bacteria was found to be dose-dependent, increasing in size with increasing amounts of ZnO from 0.5 up to 5 wt %. 

More recently, chitosan-PEG–HAp composite scaffolds were prepared including ZnO NPs [108]. The antimicrobial activity against Gram-negative *E. coli* and Gram-positive *Lysinibacillus fusiformis* and *Bacillus cereus* was evaluated, firstly for each separate component of the hybrid composite, and lastly for the overall composite system. Apart from polyethylene glycol (PEG), each single component showed promising antibacterial properties, with the maximum inhibition activity obtained from ZnO NPs against *Bacillus cereus*. The antimicrobial activities were evaluated and correlated to the presence of different amounts of nHAp-ZnO NPs. In such situations, the inhibition activity increased by increasing the weight percentage of the nanofillers, achieving the maximum activity for 15 wt % nHAp–ZnO composite. In accordance with the above-mentioned results, a more effective inhibition activity against Gram-negative bacteria was pointed out. The reason was ascribed again to the thicker cell-wall of Gram positive bacteria, which limited the penetration of the composite into the cytoplasm and its antimicrobial activity.

### 4.3. Wound Healing Applications

The antibacterial properties of ZnO-based composites also suggested their potential use for wound healing. When tissue injuries occur, the natural response of the human body first deals with an inflammatory phase. This is followed by the proliferation phase, during which wound healing effectively begins. This involves the formation of granulation tissue, which represents the ideal surrounding for angiogenesis, i.e., the formation of a new network of blood vessels. Finally, re-epithelialization and closing of the wound may take place.

Fibroblast cells play a key role in the formation of healthy granulation tissue, and hence in the promotion of injury repair. To this purpose, the adhesion and growth of fibroblasts on sodium alginate/poly(vinyl alcohol)/ZnO NP composites was investigated [112]. Beyond their antibacterial properties, these composite materials showed that fibroblasts were able to adhere and spread well after in vitro seeding for 48 h. However, a lack of cell spreading was observed as the ZnO concentration increased from 0.5 to 5 wt %. In the latter case, cells showed a rounded morphology, representative of the slight toxic effects of ZnO NPs towards fibroblast cells. This was also confirmed by cytotoxicity results that showed a decrease in cell viability with an increase of ZnO NPs amount. A concentration of 2 wt % was selected as the optimal amount for ZnO NPs to be embedded within the polymer matrix, to get at the same time the maximum antibacterial effect and the lowest cytotoxicity against fibroblasts. 

**Figure 18:**
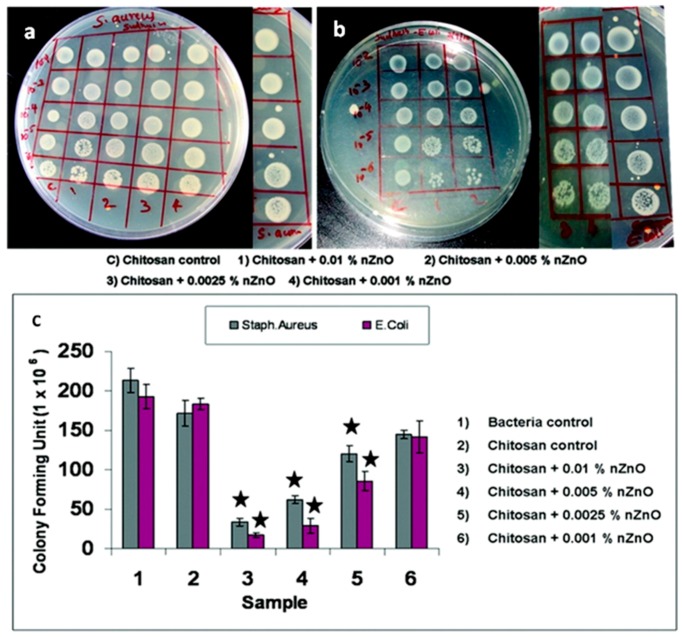
Photographs showing the antibacterial activity of composite bandages against (**a**) *S. aureus* and (**b**) *E. coli*. (**c**) Quantification of the antibacterial activity (Star symbols represent the *p* < 0.05 level, indicating that the means are significantly different, compared with the control). Adapted with permission from reference [24]. Copyright (2012) American Chemical Society.

Another study investigated both in vitro and in vivo the wound dressing and wound healing properties of chitosan hydrogel/ZnO NP composite bandages (CZBs) [24]. The authors first considered the effect of ZnO NPs on the in vitro biodegradation and antibacterial activity of CZBs. It was found that biodegradation of the overall hybrid structure was not altered by the presence of the NPs, while significant antibacterial properties against *S. aureus* and *E. coli* were achieved, as witnessed in Figure 18a,b by the appearance of inhibition zones around the doped samples during bacteria culture. Figure 18c also highlights that the antimicrobial activity was dose-dependent, being always remarkable in the presence of high amounts of ZnO NPs. The formation of ROS and Zn^2+^ release was considered the main factor in inducing antimicrobial activity, due to their aggressive action against the negatively-charged bacterial cell wall, inducing cell wall leakage and bacteria death.

**Figure 19:**
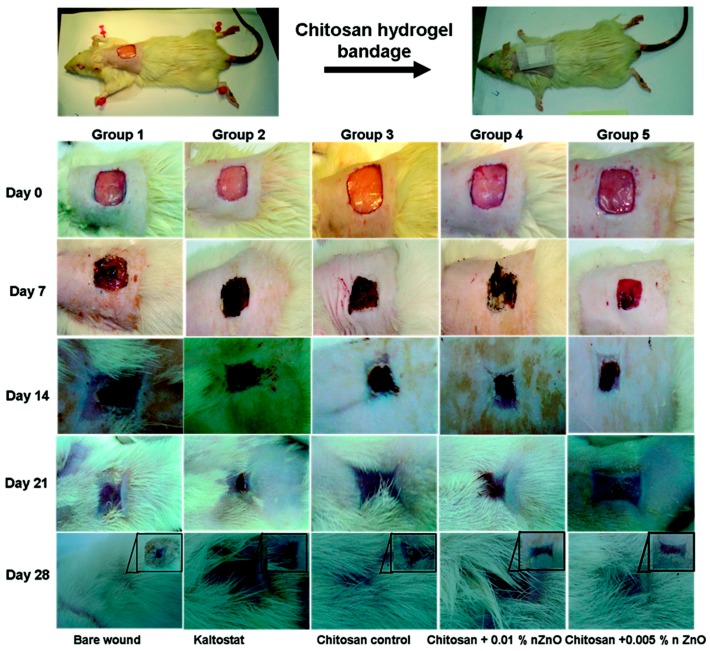
Photographs of an in vivo wound healing study. Note the extent of wound closure in the wounds treated with chitosan control and the chitosan hydrogel/ZnO NP composite bandages. Reproduced with permission from reference [24]. Copyright (2012) American Chemical Society.

Normal human dermal fibroblast (nHDF) cells were cultured both on the pure and ZnO-loaded composite bandages, showing a good level of adhesion and infiltration across the bandage structure, especially in presence of ZnO. Cell viability was found to be always in the 60–90% range after incubation for 24–48 h, independent of ZnO concentration. However, a slight decrease was observed during the first 24 h, due to the interaction between cells and ZnO NPs. Moreover, the ability of nHDF cells to attach and infiltrate well across the bandage was found to be ZnO dose-dependent; more cells were attached and infiltrated on CZBs with a lower ZnO content. Figure 19 shows the time evolution of the wound healing process obtained upon the in vivo implantation of undoped and ZnO-doped chitosan hydrogel bandages in Sprague-Dawley rats. After two weeks, wounds treated with CZBs achieved a significant closure of around 90%, while the control sample (the bare wounds and those treated with Kaltostat, a commercial bandage for wound dressing) only showed a closure of around 70%.

Moreover, after four weeks, the CZBs also resulted in a high production of densely packed keratinocytes and collagen, together with the presence of complete re-epithelialization. The addition of ZnO NPs to the chitosan hydrogel also allowed for the formation of rete-pegs in the treated wound, which indicated advanced healing of the neo-epidermidis. Furthermore, the addition of antibacterial ZnO NPs induced a strong reduction in the viability of three different bacteria that affected wounds during the in vivo experiments.

Fibroblast viability, adhesion and proliferation on ZnO-doped PU electrospun fibers was also investigated by Amna et al. [113]. The results showed that fibroblasts proliferated well on the composite fibers, without showing any stress-related cells, high attachment and proliferation. Thanks to the formation of an ultrafine mesh-like nanofiber network, cells were able to attach to the composite very well, resulting in a confluent growth of fibroblasts after five days of incubation. Moreover, the maximum number of living cells was achieved only for the ZnO-doped composite scaffold.

Beyond promoting fibroblast activity, an additional requirement in fabricating biomaterials for wound healing applications is the presence of angiogenesis properties, i.e., the ability to form new blood vessels. This aspect was explored by Bose and coworkers, by considering the effect of ZnO doping in three-dimensionally-printed calcium phosphate scaffolds [18]. After implantation in vivo of both pristine and doped β-TCP scaffolds from six weeks and up to 16 weeks, higher new blood vessel formation and more evident vascular branching morphogenesis were successfully achieved in the case of the doped samples. The observed proangiogenic properties were ascribed to the presence of Zn. Indeed, it was demonstrated that Zn induces vascularization by upregulating the release of vascular endothelial growth factor from osteoblast cells, through the increase in fibroblast growth factor production [114].

The possibility to improve fibroblast adhesion and proliferation was also successfully demonstrated for PCL/ZnO NP composite fibers [22]. Both the pristine and hybrid composite fibers were implanted in vivo in the dorsal cervical defects of guinea pigs. Then, their ability to promote fibroblast adhesion and proliferation, as well as to promote wound healing, were investigated. After 10 days of implantation, a large number of fibroblasts adhered and grew through the PCL scaffolds doped with ZnO NPs at 1 wt %. On the other hand, pristine PCL fibers showed a lower number of adhered fibroblasts. After 20 days, the PCL membrane with 1 wt % ZnO NPs showed better cell attachment and proliferation, with fibroblasts exhibiting their characteristic elongated spindle-shaped morphology. Conversely, the fibroblast cells on the pristine PCL possessed a spherical morphology. Independently of the sample, cells were able to migrate from the body wall side to the cutaneous side of the animal. However, some differences were appreciated also in this situation. After 10 days of implantation, a huge number of cells moved from the subcutaneous region towards the skin when ZnO NPs were present in the PCL matrix. On the contrary, the migration of cells was limited in the case of the pristine PCL matrix, with fibroblasts staying in the body wall side and reaching the cutaneous side only after 20 days. Therefore, ZnO NPs also promoted fibroblast migration. 

**Figure 20:**
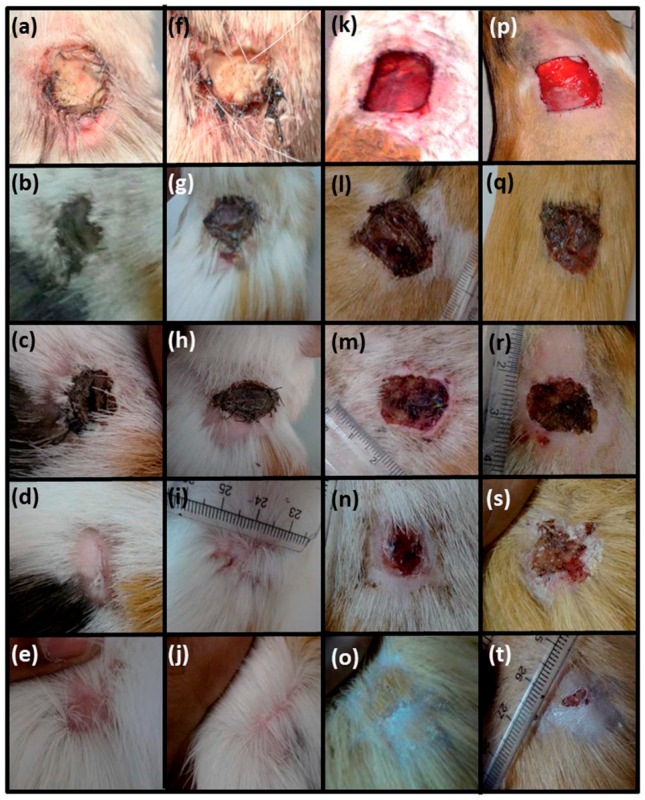
Wound healing activity of the membrane on the first day (**a**,**f**,**k**,**p**); on the fifth day (**b**,**g**,**l**,**q**); on 10th day (**c**,**h**,**m**,**r**); on 20th day (**d**,**i**,**n**,**s**) and on 30th day (**e**,**j**,**o**,**t**) of implantation. The first column (**a**–**e**) indicates neat polycaprolactone (PCL) membranes; the second column (**f**–**j**) indicates PCL membrane incorporated with 1 wt % ZnO nanoparticles; the third column (**k**–**o**) indicates povidone–iodine treated wounds (positive controls) and the fourth column (**p**–**t**) indicates negative controls. Reproduced with permission from reference [22]. Copyright (2014) Royal Society of Chemistry.

Figure 20 summarizes the wound healing properties obtained for both pristine PCL and PCL/ZnO NPs (1 wt %) membranes sutured along the whole thickness wound in guinea pigs. During the first days, the wounds were fully covered by the membranes, thereby making difficult any evaluation of the healing process. Then, after 15–20 days of implantation, both the pristine and ZnO-doped PCL membranes were expelled from the healing wound. Here, a noticeable difference in healing was clearly observed, with wounds treated with ZnO-doped PCL membranes being completely healed (panel j of Figure 20). The rate of wound healing was then evaluated, by estimating the area of healed wound until the wound was completely healed. Even though there was no significant differences after 10 days of healing between pristine and ZnO-doped PCL membranes, ZnO-doped PCL showed a higher rate of wound healing, achieving 100% after 25 days. On the contrary, for the pristine PCL membrane the wound was still not completely healed at the end of the study (panel e of Figure 20). The promising behavior observed for the ZnO-doped PCL matrix was mainly due to the ability of ZnO NPs in promoting ROS generation, H_2_O_2_ in particular, which has been demonstrated to play a fundamental role in upregulating the expression of important angiogenic mediators like VEGF and fibroblast growth factor-2 (FGF2).

The same research group also investigated the proangiogenic properties of ZnO-doped PCL fibers [23]. A chicken chorioallantoic membrane (CAM) assay was performed to evaluate the formation of new blood vessels. In the presence of 1 wt % ZnO NPs, the composite PCL membranes were able to significantly increase the formation of large matured blood vessels, together with the rise of a highly branched capillary network. However, the authors also found that the angiogenic properties of the doped fibers were dependent on the amount of NPs. For ZnO NPs equal to 0.5, 2 and 4 wt %, the number of branching points in the newly-formed blood vessels was significantly lower than those observed for PCL fibers doped with 1 wt % ZnO NPs. 

**Figure 21:**
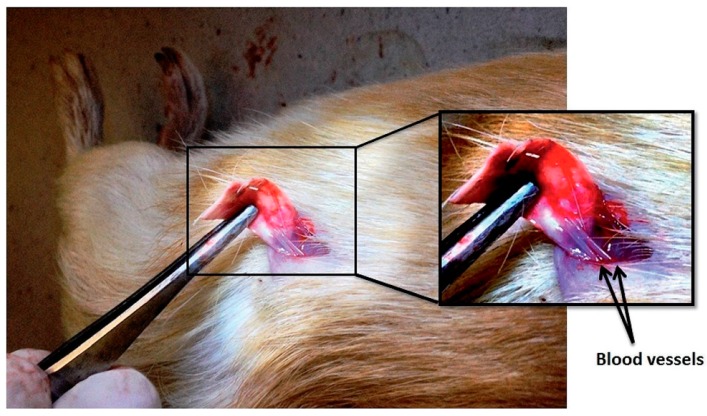
Matured blood vessel through the implanted polycaprolactone scaffolds containing 1 wt % ZnO nanoparticles after 20 days of subcutaneous implantation. Reproduced with permission from reference [23]. Copyright (2014) Royal Society of Chemistry.

**Figure 22:**
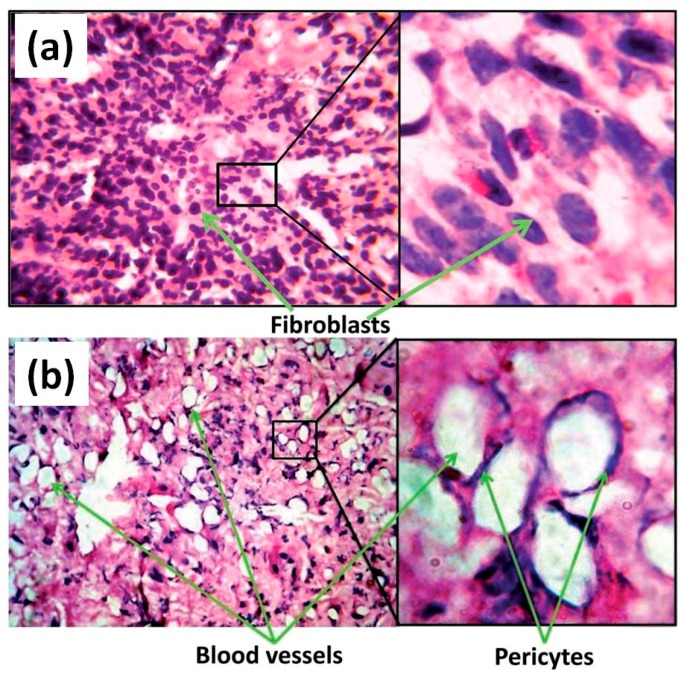
Formation of well-defined vasculature after 20 days of subcutaneous implantation of polycaprolactone (PCL) scaffolds containing 1 wt % ZnO nanoparticles (**b**); (**a**) indicates neat PCL scaffolds without vasculature (light microscopic image after staining with Hematoxylin & Eosin, 100× magnification). Insets show enlarged portions, taken at 1000× magnification. Modified with permission from reference [23]. Copyright (2014) Royal Society of Chemistry.

The proangiogenic properties of PCL-1 wt % ZnO NP membranes were also investigated in vivo. After five days of subcutaneous implantation in guinea pigs, angiogenesis was observed for the doped PCL membrane. In this case, a large number of red blood cells migrated towards the membrane, coupled with a lot of fibroblasts migrating from the sides of the scaffold to the interior part. Then, the formation of two large blood vessels, showed in Figure 21 and passing through the subcutaneously implanted membrane, were also visible after 20 days of implantation. The sectional view of these vessels further underlined the significant proangiogenic properties of the ZnO-doped PCL scaffold, where the presence of the extensive vascular network throughout it and of perycites around vessels were clearly visible, as shown in Figure 22. All these aspects were not observed for the pristine PCL scaffold.

More recently, three-dimensional nanostructured porous granules of HAp and ZnO NPs were also considered in view of their antibacterial, biocompatible and angiogenic abilities [21]. The nano-HAp granules with and without ZnO NPs were implanted in vivo into the subcutaneous tissue in rats. After three, seven and 30 days of implantation, the inflammatory response was examined. It was observed that the developed composites effectively reduced bacterial activity for an optimal ZnO percentage of 2 wt %. More strikingly, the intense formation of new vascularization around the granules after three days of implantation was noticed. Then, high-density connective tissue together with the presence of fibroblasts and mature blood vessels was present after 30 days.

Novel electrospun polyvinilidene-fluoride-trifluoroethylene (P(VDF-TrFE))/ZnO NP nanocomposites were also recently investigated with the aim of guiding new tissue regeneration due to the piezoelectric properties of the composite components [115]. The authors first demonstrated the biocompatibility of the nanocomposite system by culturing human mesenchymal stem cells (hMSCs) and HUVECs. It was found that higher cell viability, adhesion and proliferation compared to cells cultured on pristine P(VDF-TrFE) were obtained in vitro. The presence of angiogenic properties was also successfully proved in vivo, after subcutaneous implantation in the abdominal region of Wistar rats for seven and 21 days. A highly branched vasculature was observed especially in the case of P(VDF-TrFE)/ZnO scaffolds pre-seeded with hMSCs. Extensive networks of collagen fibers throughout the whole scaffold were noticed after seven days of implantation, together with the neo-vascularization of the tissues around the scaffold and with an amount of newly formed blood vessels dependent on the ZnO percentage incorporated within the nanocomposite. After 21 days, the in-growth of blood vessels was further confirmed. Also in this work, the observed angiogenic properties were due to the presence of ROS generated from ZnO NPs.

## 5. Conclusions

In summary, ZnO nanostructures alone or combined with ceramic and polymer matrices are emerging as promising materials for tissue engineering. Pure ZnO structures demonstrated good antibacterial properties and the ability to significantly support the growth, adhesion and proliferation of several cellular lines, with a special affinity for osteoblast-like cells. Such amazing results were mainly due to the combination of both nanotopographic effects, ZnO piezoelectricity and the osteogenic/antibacterial action of zinc ions. On the other side, hybrid composite scaffolds incorporating ZnO in different amounts turned out to be a strategic tool for the formation of new bone tissue, as well as in the promotion of newly-formed blood vessels for wound healing. In several cases, osteogenesis and angiogenesis due to ZnO were successfully combined with the use of additive manufacturing technologies to design novel, advanced scaffolds for tissue engineering, and their unique properties demonstrated by in vitro and in vivo experiments. While the ability to induce new bone formation was generally ascribed to the intrinsic osteogenic activity of Zn, the generation of ROS due to ZnO was reported as the main reason for the observed angiogenic properties. New generation scaffolds for bone TE and wound healing applications could thus be envisioned by the combination of osteogenic and angiogenic ZnO nanostructures, coupled to their antibacterial and piezoelectric behavior. Even though encouraging results have been reported, several aspects need to be further investigated. Some examples cover the specific role played by ZnO piezoelectricity in driving cell proliferation and the effect of ROS in stimulating the formation of new blood vessels. The further unraveling of these working mechanisms would open up the way to successful in vivo studies and clinical trials.
